# Where Are We on the Animal Welfare Map? Using GIS to Assess Stakeholder Diversity and Inclusion

**DOI:** 10.3389/fvets.2021.785071

**Published:** 2021-12-13

**Authors:** Kevin C. Roberts, Tegan L. Buckingham, Kyrsten J. Janke, Linda S. Jacobson

**Affiliations:** Toronto Humane Society, Toronto, ON, Canada

**Keywords:** animal welfare, diversity, inclusion, geographic information system, GIS, stakeholders, Ontario marginalization index

## Abstract

Inequities exist in all facets of society, and animal welfare organizations (AWOs) and their communities are no exception. These organizations interface with multiple stakeholder groups. An active analysis of stakeholder groups to identify under-served areas and communities has not been performed. Using stakeholder data from Toronto Humane Society (THS) from 2015–2019, this study performed a retrospective spatial analysis to identify well served and under-served geographic areas for adopters, surrenders, public veterinary service (PVS) clients, volunteers and foster parents, using Hot Spot analysis. Correlation analysis was performed to determine whether the spatial distribution of the groups correlated with the four socioeconomic metrics of the 2016 Ontario Marginalization Index (residential instability, material deprivation, dependency, and ethnic concentration), and a metric representing the distribution of Indigenous residents. For each stakeholder group, there were well served areas, typically in central Toronto where THS is located, and under-served areas, typically in the north-west and north-east corners of Toronto and in the surrounding cities of the Greater Toronto Area. The area served by THS PVS extended further north than the other hot spot areas. The number of adopters increased as the residential instability metric increased, whereas the number of adopters decreased as the ethnic concentration metric increased. The rate of surrenders increased as the Indigenous metric increased. Public Veterinary Service clients increased as the residential instability, material deprivation, and Indigenous metrics increased. One of the primary limitations of this study was the confounding factor of distance from THS. Individuals living further from THS are less likely to utilize its services, particularly if there is another accessible AWO nearby, and therefore may appear to reflect an under-served population that may not truly be under-served. A regional approach would help to overcome this limitation. The results provide useful insights into stakeholder engagement and provide a foundation for analysis of more targeted areas, as well as for strategies to reach under-served demographics. Similar analyses by other AWOs would be helpful to address inequities in a larger geographic area. Animal welfare organizations can improve program effectiveness by adding data analytics skills to the more traditional skills associated with this sector.

## Introduction

The COVID-19 pandemic has exposed and exacerbated deep and chronic societal inequities, many of which are directly related to race and class (1). The murder of George Floyd in May 2020 was the catalyst for sustained protests by Black Lives Matter and other movements, primarily in the U.S. but also in other countries, including Canada. This defining moment has led to seismic changes in social awareness, causing many individuals and organizations to examine their role and culpability in perpetuating systemic inequities, and their responsibility to acknowledge and address past mistakes. This has prompted many animal welfare organizations (AWOs) to consider the role of diversity, equity, and inclusion (DEI) in the design of support services and general operations (2). Since the start of the pandemic, AWOs have increasingly focused on supporting the human-animal bond by providing and promoting support for both pet parents and their pets, similar to traditional social service supports^1^ (3). Additionally, new organizations have formed to promote inclusivity and combat biases^2^. Animal welfare organizations interface with, and create a community of, multiple stakeholder groups. These include those who derive meaning and belonging from giving (volunteers, foster parents), those who welcome and cherish new non-human family members (adopters), and those who utilize other services provided by AWOs (surrenders, public veterinary services). This culture of compassion and giving is difficult to reconcile with the statement that “the animal welfare industry lives at the intersection of white privilege and systemic racism” (4).

Conversely, stakeholders include those who may be punished by and discriminated against by existing systems^3^^,^^4^. Strays and surrenders are the largest sources of shelter intakes (75–80%) (5). Members of some communities are disproportionately more likely to surrender animals to AWOs, for reasons that are often directly related to poverty and social vulnerability (6, 7), or to be declined for adoption (8). In some jurisdictions, low-income and racialized individuals may be fined for their inability to comply with local ordinances such as compulsory spay/neuter (8). In one study, pet parents with lower income and less education were less likely to be able to find their lost pets (9). While many AWOs and some community clinics provide free or low-cost veterinary care, in particular vaccination and spay/neuter (10), these services are only available to those who are aware of them, understand their benefits, and have physical and financial access to them (11, 12).

Community-driven organizational activities require inclusion and representation of the community within organizations providing these services. Stakeholders such as volunteers and foster parents, who benefit from participating in and supporting organizational activities, may not represent the diversity of the community being served. Low-income and racialized communities may be overlooked in fundraising drives and searches for new volunteers, despite the fact that members of these communities have the means and desire to participate (13). Animal welfare organization staff in the U.S. and Canada are overwhelmingly white (14). Historically, the sector has not prioritized training around effective, non-judgmental engagement with non-English-speaking immigrants and marginalized and vulnerable individuals and communities. Animal welfare organizations, especially those with animal control responsibilities, can be seen as unwelcoming and threatening authority figures (3).

There is little Canadian data regarding social justice and equity in the animal welfare sector.

Passive or non-existent stakeholder analysis could perpetuate inequities and limit the effectiveness of support services. In contrast, an active analysis of service gaps and stakeholder composition would allow for strategically targeted remediation in under-served areas. In June 2020, Toronto Humane Society (THS), an independent charitable AWO in downtown Toronto, Canada, published a statement^1^ in support of Black Lives Matter, and committed to specific actions to redress inequities. One of these commitments was to examine the spatial patterns of different stakeholder groups served by the organization.

The main objective of this study was to use geographic information systems (GIS) to identify and analyze geographic areas and communities currently under-serviced by our organization, in order to create targets for future programs and interventions. Geographic information systems are computer systems that are used for the creation, storage, analysis, and mapping of digital data. A secondary objective was to develop a robust methodology for the project and share this with other AWOs.

## Materials and Methods

### Study Area

The study analyzed data from the Greater Toronto Area (GTA) in Ontario, Canada. The GTA is the most populated metropolitan area in Canada and includes the city of Toronto and the regional municipalities of Halton, Peel, York, and Durham. As of 2016, the population was 5,928,040 (15). The GTA is comprised of 1,274 census tracts (CTs) of varying sizes, with populations ranging from 10–23,401. According to the 2016 Census of Canada (15), approximately 15% of the population of the GTA are considered low-income, as classified by the “low-income measure after tax” metric. Approximately 51% of the population identify as racialized, and 0.7% identify as Indigenous (15). Toronto Humane Society is centrally located in the city of Toronto and aims to serve the entire GTA, and in some cases, communities beyond the GTA borders. The scope of this research was restricted to THS stakeholders within the GTA.

### Data

#### Stakeholder Data

Data from 2015–2019 were extracted from THS' PetPoint shelter management and Volgistics volunteer management databases, and retrospectively analyzed. The programs included in this study were surrender, stray intake, adoption, foster care, and public veterinary services. Public veterinary services included (but were not limited to): spay-neuter surgery, vaccinations, preventative wellness, dentistry and owner-requested euthanasia. Stakeholders were divided into five groups: adopters, surrenders, public veterinary service (PVS) clients, volunteers, and foster parents.

Prior to data cleaning, stakeholder group sizes were: adopters *n* = 16,133, surrenders *n* = 18,479, PVS clients *n* = 59,204, volunteers *n* = 2,020, foster parents *n* = 5,522. Instances of a single stakeholder appearing multiple times within the same year in the same group were removed. After removing these duplicates, stakeholder group sizes were: adopters *n* = 14,464, surrenders *n* = 6,647, PVS clients *n* = 33,740, volunteers *n* = 1,990, foster parents *n* = 2,146. Data cleaning was then performed to exclude stakeholders located outside the GTA, and those who did not provide a complete home address and could not be geocoded. The remaining data was geocoded using the MMQGIS Geocode plugin QGIS, and the resulting points were projected onto the Esri world topographic map (16). The final stakeholder group sizes that were successfully geocoded and used in the analysis were: adopters *n* = 13,837, surrenders *n* = 5,740, PVS clients *n* = 31,074, volunteers *n* = 1,989, and foster parents *n* = 2,054.

#### Ontario Marginalization Index

The study utilized the 2016 Ontario Marginalization Index (ON-Marg), which was developed jointly by Public Health Ontario and the St. Michael's Hospital Center for Urban Health Solutions to measure marginalization in CT areas (17). The Index utilizes a combination of 18 indicators to define four distinct metrics representing marginalization. These are: (1) residential instability, (2) material deprivation, (3) dependency, and (4) ethnic concentration (Table 1).

**Table 1:** The four metrics of marginalization comprising the 2016 Ontario Marginalization Index (17).

**Metric**	**Description**	**Indicators**
Residential Instability	This measure refers to people who experience high rates of family or housing instability. Indicators focus on the type and density of residential accommodations, as well as certain family structure characteristics.	• Proportion of the population living alone • Proportion of the population who are not youth (age 5–15 years) • Average number of persons per dwelling • Proportion of dwellings that are apartment buildings • Proportion of the population who are single/divorced/widowed • Proportion of dwellings that are not owned • Proportion of the population who moved during the past 5 years
Material Deprivation	This measure relates closely to low income levels and refers to the individual and community's inability to access and attain basic material needs.	• Proportion of the population aged 20+ without a high-school diploma • Proportion of families who are single parent families • Proportion of total income from government transfer payments for population 15+ • Proportion of the population aged 15+ who are unemployed • sProportion of the population considered low-income • Proportion of households living in dwellings that are in need of major repair
Dependency	This measure refers to people who do not have employment income, including children, adults, and seniors whose work is not compensated.	• Proportion of the population aged 65+ • Dependency ratio (total population 0-14 and 65+/total population aged 15-64) • Proportion of the population not participating in the labor force
Ethnic Concentration	This measure refers to people who are recent immigrants and those who self-identify as being members of a racialized community (not including Indigenous peoples).	• Proportion of the population who are recent immigrants (arrived in the past 5 years) • Proportion of the population who self-identify as being part of a racialized community

#### Indigenous Populations

Indigenous indicators are not included in ON-Marg because of undercounting of Indigenous communities in the Canadian Census (19). To compensate for this, we included a normalized Indigenous population metric, namely the number of Indigenous residents per 1,000 total residents in each CT, based on data from the 2016 Census of Canada (15).

### Analysis

#### Spatial Cluster Hot Spot and Cold Spot Analysis

Prior to analysis, a spatial join was performed on the geocoded data for each of the five stakeholder groups, to join them to a map of the CT boundaries. This created a field within the CT attribute table containing a count of the number of stakeholder points falling within each CT area (Supplementary Figures 1–5). The count variable for each stakeholder group was then normalized to population size (1,000 ^*^ count variable/2016 population), to account for variations in CT population potentially skewing the results (Supplementary Figures 6–10). The normalized rates were used throughout the analysis.

Hot spots were defined as statistically significant areas of high stakeholder density surrounded by other areas of high stakeholder density. Cold spots were statistically significant areas of low stakeholder density surrounded by other areas of low stakeholder density (20). To locate statistically significant hot spot and cold spot clusters, the Getis-Ord Gi^*^ statistic was calculated using the Optimized Hot Spot Analysis tool in ArcGIS Pro version 2.8 (20–22).

#### Correlation Analysis

Statistical correlation analysis was conducted to identify statistically significant relationships between the five stakeholder groups (dependent variables) and the four ON-Marg metrics, as well as the additional Indigenous population metric (explanatory variables).

To account for instances of false positives caused by multiple testing and spatial dependency within the data, a false detection rate correction was implemented in both the spatial cluster hot spot and cold spot analysis, and the correlation analysis. The correction estimates the number of expected false positives for a given confidence interval and adjusts the critical *p* value accordingly, effectively removing the weakest statistically significant results^5^.

Spatial correlation was determined using the Local Bivariate Relationship tool in ArcGIS Pro version 2.8 (22), with significance set at *p* < 0.05. The tool classifies the relationship as one of six types defined in Table 2. The convex and concave relationships identified by the tool are not necessarily symmetrical curves and may reflect primarily negative or positive associations (Table 2). When a statistically significant spatial correlation was detected between a stakeholder group and one of the tested metrics, the result was generated as the percentage of the total number of CTs having a significant relationship, and then broken down to the percentage of CTs having each type of significant relationship. This analysis reflects the association between the dependent and explanatory variable for each CT. This differs from the hot spot and cold spot analysis, which reflects the number of stakeholders in a CT compared with surrounding CTs. Where a statistically significant relationship was detected between a stakeholder group and one of the tested metrics, these results were quantified as the percentage of the total number of CTs (*n* = 1,274) having that type of relationship.

**Table 2:** The six relationship types defined by the Local Bivariate Relationship tool in ArcGIS (23).

**Relationship**	**Definition**	**Examples (see text footnote 7)**
Not significant	There is no significant relationship between the variables.	NA
Positive linear	The dependent variable increases linearly as the explanatory variable increases.	Conventional linear curve
Negative linear	The dependent variable decreases linearly as the explanatory variable increases.	Conventional linear curve
Concave	The dependent variable forms a concave curve as the explanatory variable increases. While the explanatory variable values are low, they form a positive relationship with the dependent variable, but as they increase, the relationship inverts and they then form a negative relationship with the dependent variable.	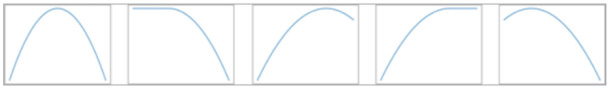
Convex	The dependent variable forms a convex curve as the explanatory variable increases. While the explanatory variable values are low, they form a negative relationship with the dependent variable, but as they increase, the relationship inverts and they then form a positive relationship with the dependent variable.	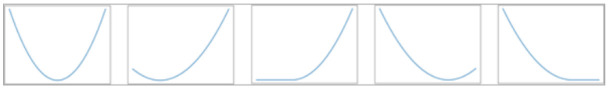
Undefined complex	The two variables are significantly related, but the nature of the relationship is different from any of the other defined relationship types.	Variable, do not fit conventional curves

*NA, not applicable*.

## Results

**Figure 1:**
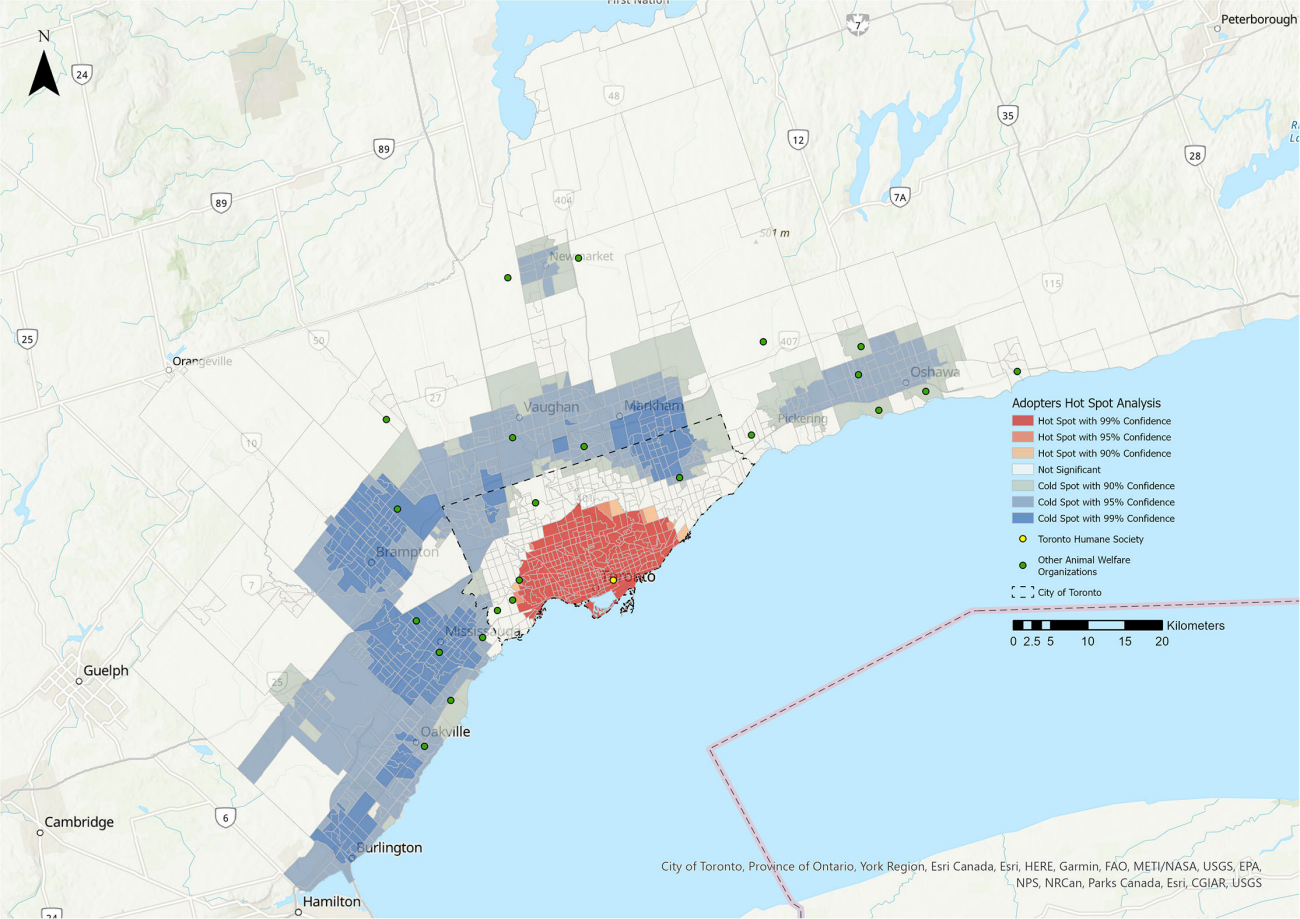
Hot Spot Analysis for the adopters stakeholder group. Shades of blue represent cold spot areas (statistically significant low number of stakeholders), and shades of red represent hot spot areas (statistically significant high number of stakeholders). Toronto Humane Society is represented by the yellow dot, and other animal welfare organizations in the Greater Toronto Area are represented by green dots.

There were 1,274 CTs in the study area. Figure 1 shows the area included and the location of THS and other GTA AWOs. Stakeholder group sizes were as follows: adopters, *n* = 13,837; surrenders, *n* = 5,740; PVS clients, *n* = 31,074; volunteers, *n* = 1,989; and foster parents, *n* = 2,054.

### Hot Spot Analysis (HSA)

**Figure 2:**
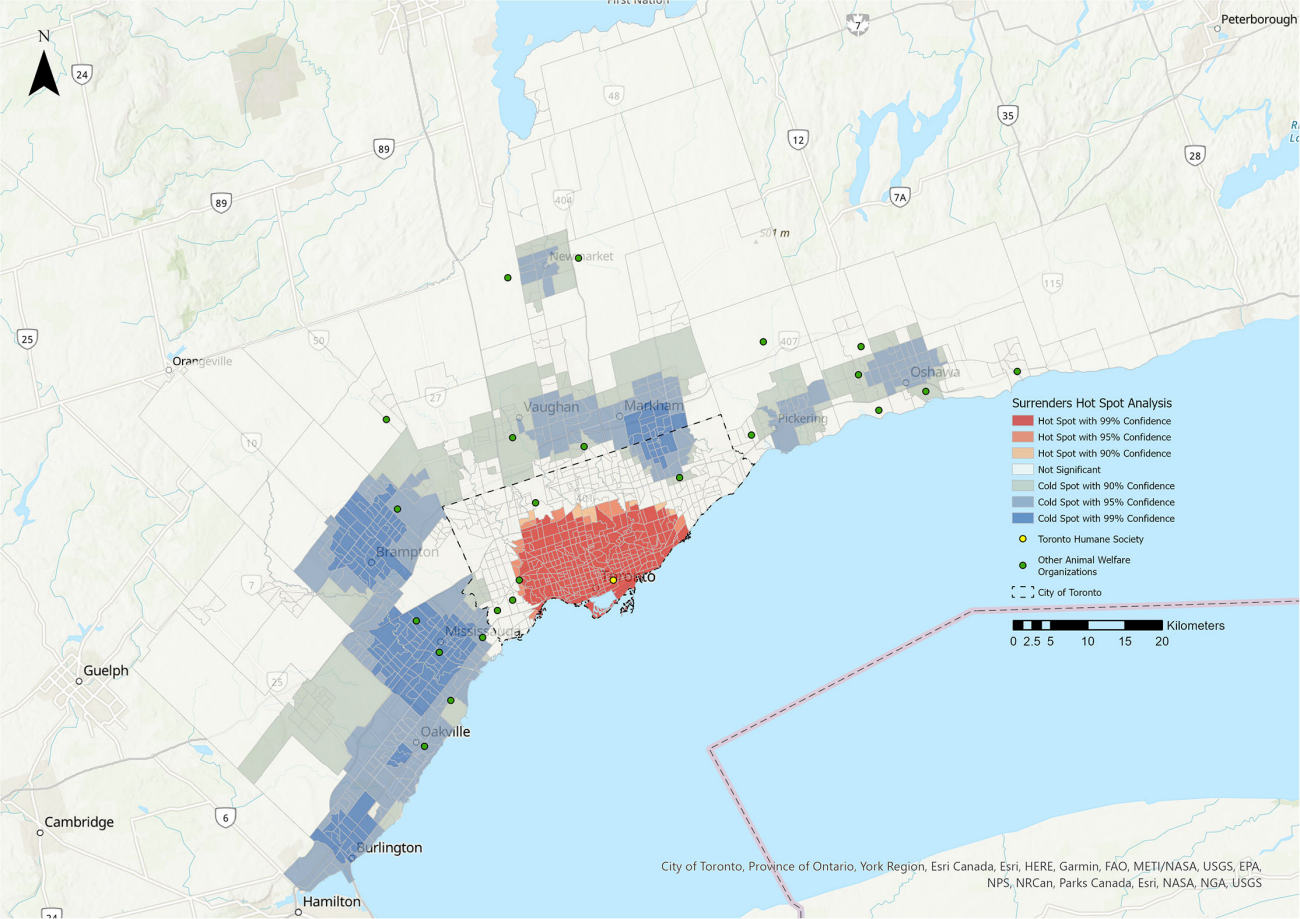
Hot Spot Analysis for the surrenders stakeholder group. Shades of blue represent cold spot areas (statistically significant low number of stakeholders), and shades of red represent hot spot areas (statistically significant high number of stakeholders). Toronto Humane Society is represented by the yellow dot, and other animal welfare organizations in the Greater Toronto Area are represented by green dots.

**Figure 3:**
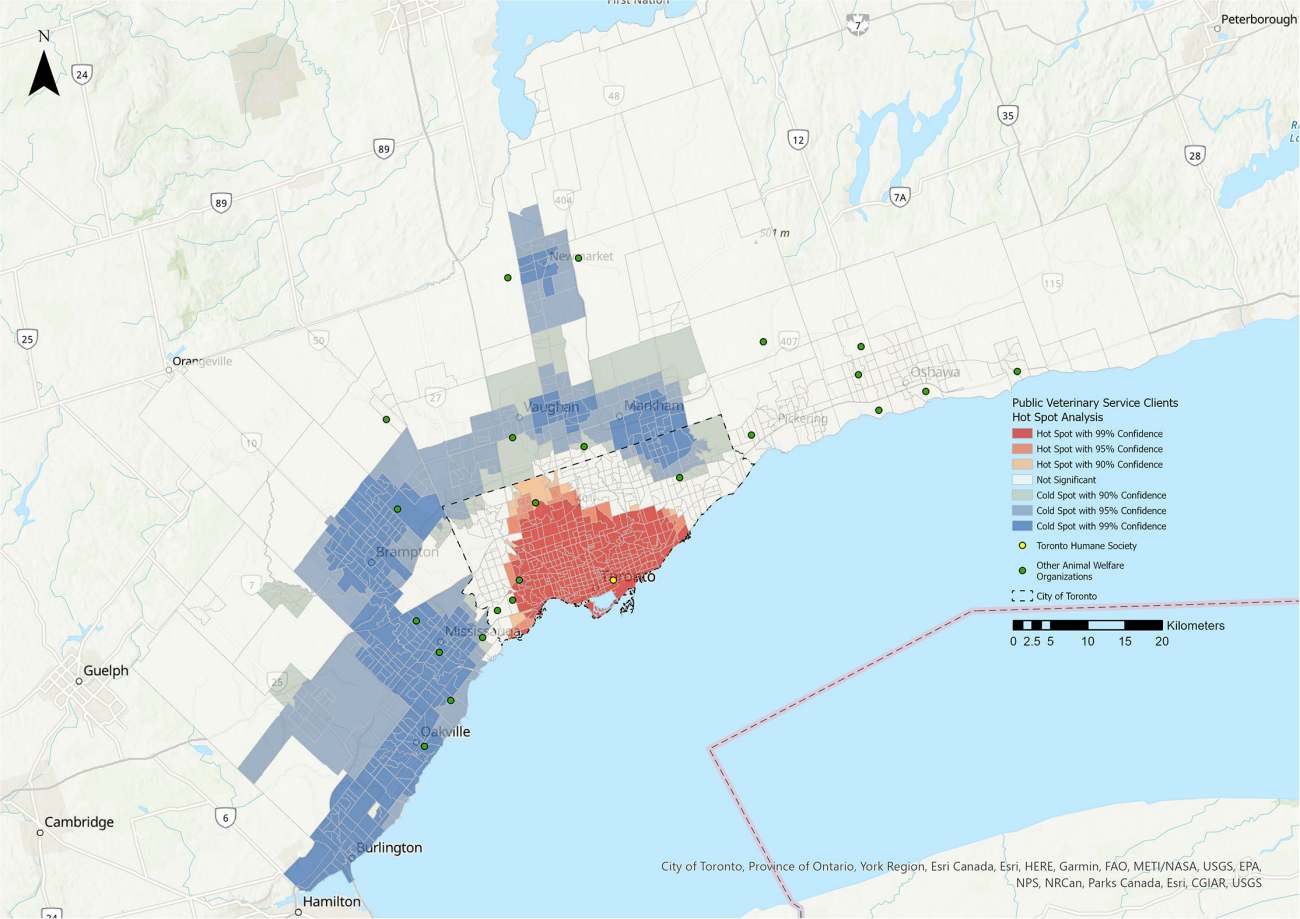
Hot Spot Analysis for the public veterinary service clients stakeholder group. Shades of blue represent cold spot areas (statistically significant low number of stakeholders), and shades of red represent hot spot areas (statistically significant high number of stakeholders). Toronto Humane Society is represented by the yellow dot, and other animal welfare organizations in the Greater Toronto Area are represented by green dots.

**Figure 4:**
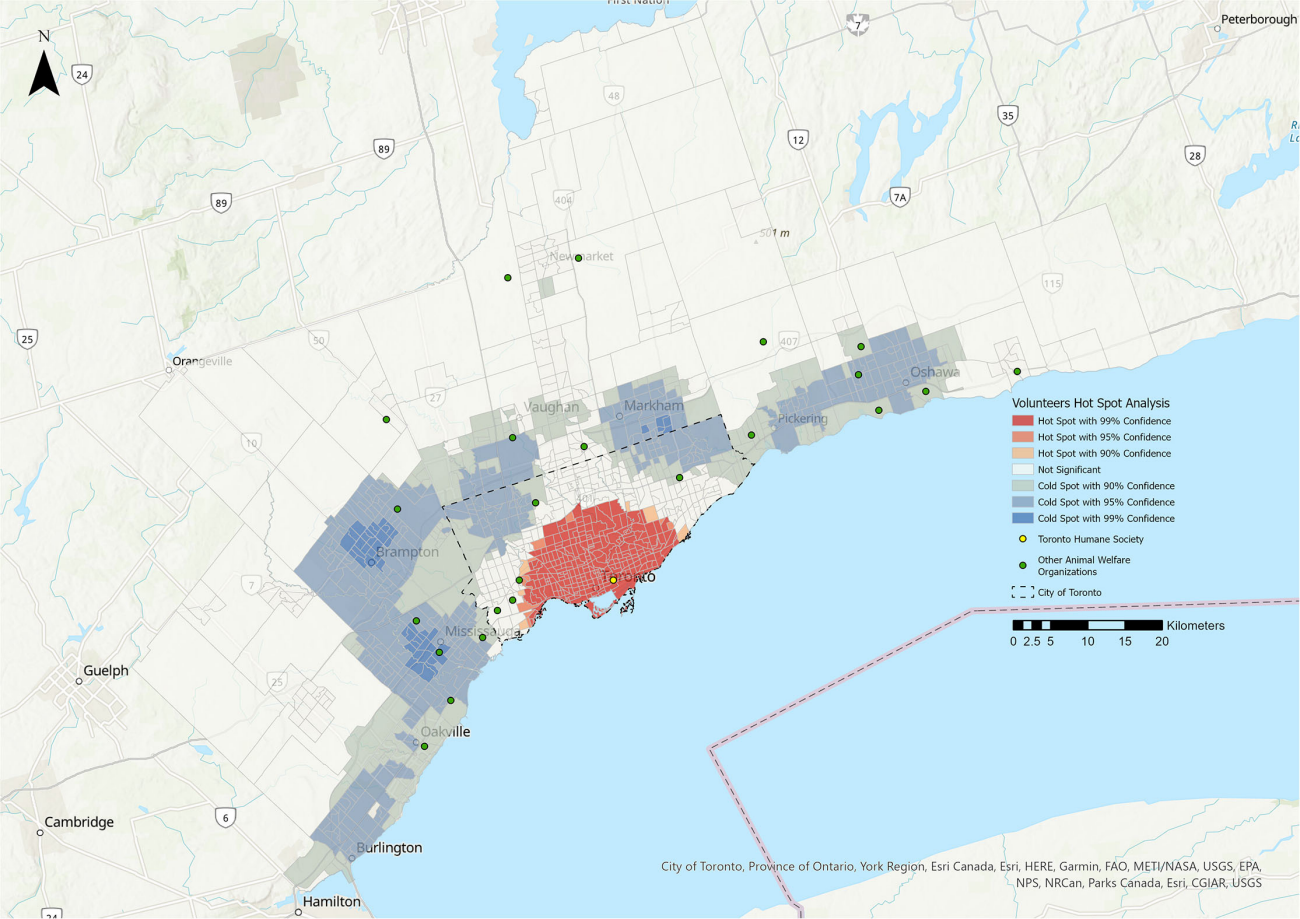
Hot Spot Analysis for the volunteers stakeholder group. Shades of blue represent cold spot areas (statistically significant low number of stakeholders), and shades of red represent hot spot areas (statistically significant high number of stakeholders). Toronto Humane Society is represented by the yellow dot, and other animal welfare organizations in the Greater Toronto Area are represented by green dots.

**Figure 5:**
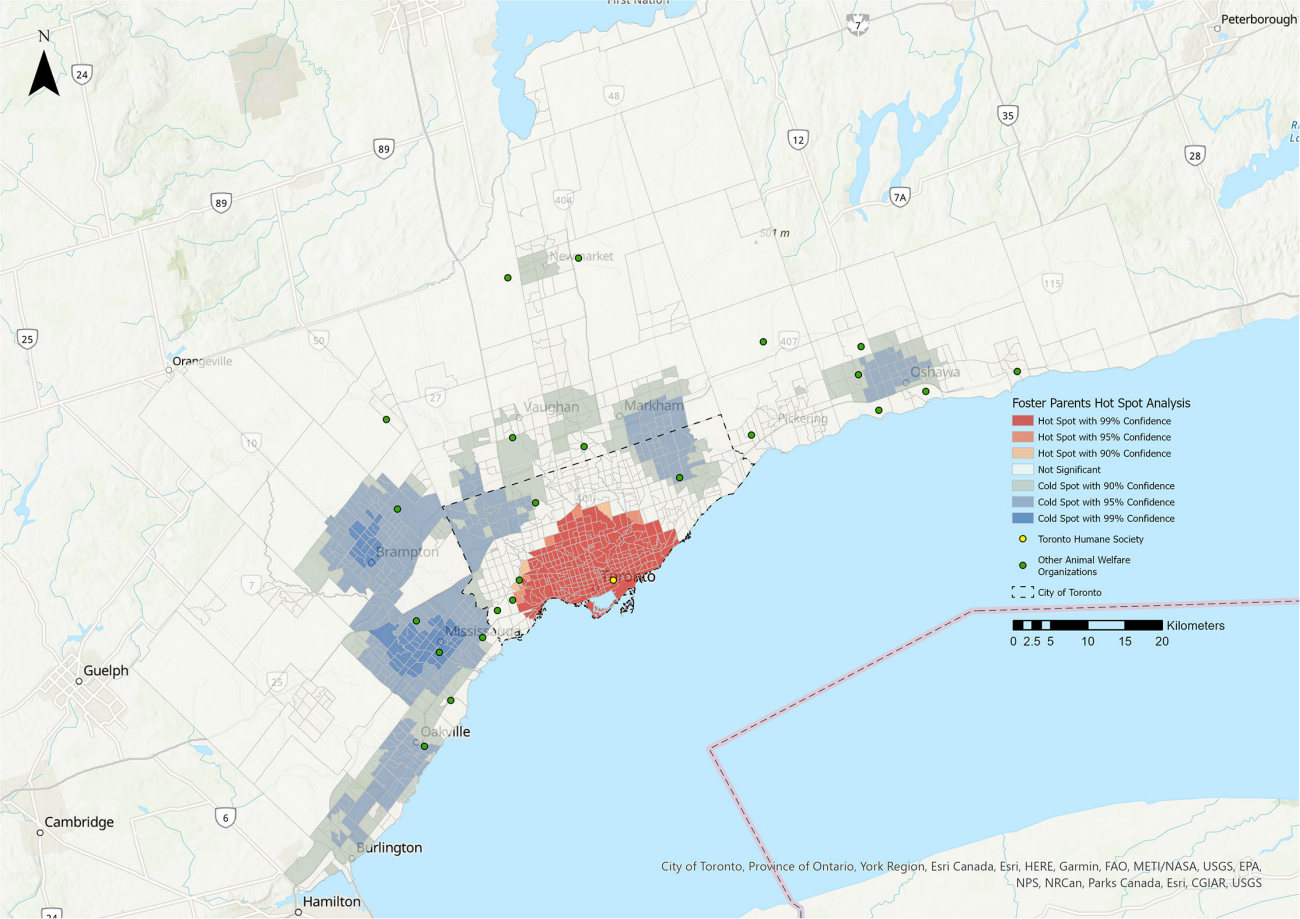
Hot Spot Analysis for the foster parents stakeholder group. Shades of blue represent cold spot areas (statistically significant low number of stakeholders), and shades of red represent hot spot areas (statistically significant high number of stakeholders). Toronto Humane Society is represented by the yellow dot, and other animal welfare organizations in the Greater Toronto Area are represented by green dots.

The results of the HSA are shown in Figures 1–**5**. All five stakeholder groups formed statistically significant hot spot clusters within the city of Toronto. The adopters, surrenders, volunteers, and foster parents formed hot spot clusters in the central region of Toronto, covering a similar geographic area (Figures 1, 2, **4**, **5**), whereas PVS clients formed hot spots with a larger geographic area reaching further north within Toronto (Figure 3).

The cold spots were more varied in their distributions. The adopters group formed cold spot clusters in the north-west and north-east corners of the city of Toronto, as well as in the surrounding cities of Burlington, Oakville, Mississauga, Brampton, Vaughan, Markham, Newmarket, Pickering, and Oshawa (Figure 1). Cold spots for the surrenders group were broadly similar in their distribution within the city of Toronto and the surrounding cities, but did not include the cluster in the north-west corner of Toronto that was apparent for adopters, volunteers and foster parents (Figure 2). Cold spots for PVS clients were also similar in their distribution in most of the surrounding cities, but clustering to the east, over the cities of Pickering and Oshawa, was absent (Figure 3). The volunteers group again formed cold spot clusters similar to those of the adopters group, with the exception of the area north of Toronto and west of Markham (Figure 4). Lastly, cold spot clusters identified in the foster parent group were more sparsely distributed than in other groups, with no clusters between the cities of Oshawa and Markham, or Markham and Brampton (Figure 5).

### Correlation Analysis

A full breakdown of the correlation classification results can be found in Table 3.

**Table 3:** Relationships between the stakeholder groups of the Toronto Humane Society, 2016–2019, and the five metrics analyzed.

**Stakeholder group** **Dependent variable)**	**Metric (Explanatory variable)**	**Relationship type (% of total features)**				
		**PL**	**NL**	**CC**	**CV**	**UC**
Adopters	Residential instability Material deprivation dependency Ethnic concentration Indigenous	**15.38%** **1.41%** **0.39%** **0.08%** **16.37**	0.00% **4.08%** **2.82%** **14.99%** 0.00%	**2.59%** 0.00% 0.08% **1.65%** **5.17%**	**3.85%** **0.86%** **2.04%** **8.56%** **0.94%**	**0.31%** **2.28%** **0.86%** **4.79%** **0.78%**
	Residential instability Material deprivation dependency Ethnic concentration Indigenous	0.00% 0.00% 0.00% 0.00% **10.18%**	0.00% 0.00% 0.00% 0.00% 0.00%	0.00% 0.00% 0.00% 0.00% **0.55%**	0.00% 0.00% 0.00% 0.00% **2.58%**	0.00% 0.00% 0.00% 0.00% **0.86%**
Public veterinary Service clients	Residential instability Material deprivation dependency Ethnic concentration Indigenous	**39.95%** **11.15%** 0.00% 0.00% **28.35%**	0.00% 0.00% 0.00% 0.00% 0.00%	**2.90%** **1.18%** 0.00% 0.00% **6.19%**	**7.61%** **0.63%** 0.00% 0.00% **8.07%**	**2.35%** **1.73%** 0.00% 0.00% **3.45%**
Volunteers	Residential instability Material deprivation dependency Ethnic concentration Indigenous	0.00% 0.00% 0.00% 0.00% 0.00%	0.00% 0.00% 0.00% **0.55%** 0.00%	0.00% 0.00% 0.00% 0.00% 0.00%	0.00% 0.00% 0.00% **1.33%** 0.00%	0.00% 0.00% 0.00% **0.16%** 0.00%
Foster parents	Residential instability Material deprivation dependency Ethnic concentration Indigenous	0.00% 0.00% 0.00% 0.00% 0.00%	0.00% 0.00% 0.00% 0.00% 0.00%	0.00% 0.00% 0.00% 0.00% 0.00%	0.00% 0.00% 0.00% 0.00% 0.00%	0.00% 0.00% 0.00% 0.00% 0.00%

*Bold type indicates statistically significant relationships*.

*Significant percentage of the total number of features (census tracts, *n* = 1,274) identified as having statistically significant relationships between the stakeholder groups and each of the five metrics. PL, positive linear; NL, negative linear; CC, concave; CV, convex; UC, undefined complex*.

#### Adopters

**Figure 6:**
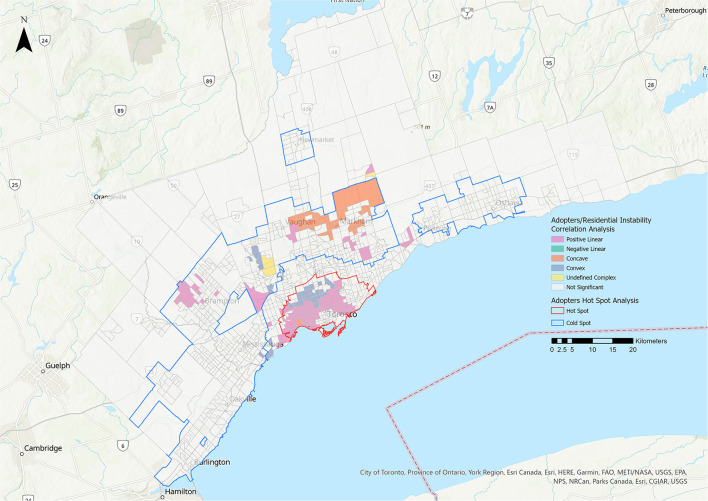
Correlation analysis for the adopters stakeholder group and the residential instability metric of the 2016 Ontario Marginalization Index. Pink represents positive linear relationships, green represents negative linear relationships, orange represents concave relationships, blue represents convex relationships, and yellow represents undefined complex relationships.

**Figure 7:**
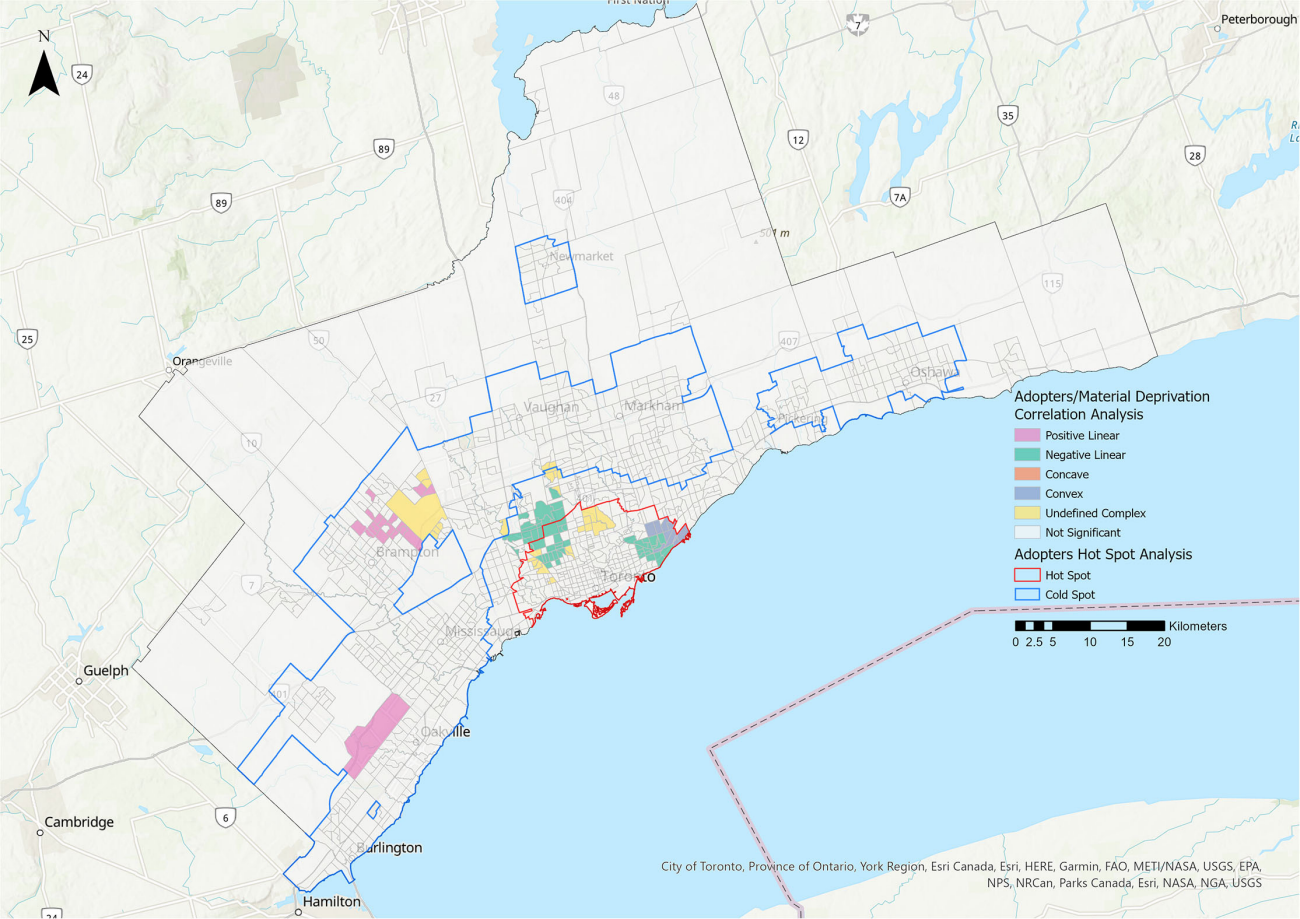
Correlation analysis for the adopters stakeholder group and the material deprivation metric of the 2016 Ontario Marginalization Index. Pink represents positive linear relationships, green represents negative linear relationships, orange represents concave relationships, blue represents convex relationships, and yellow represents undefined complex relationships.

**Figure 8:**
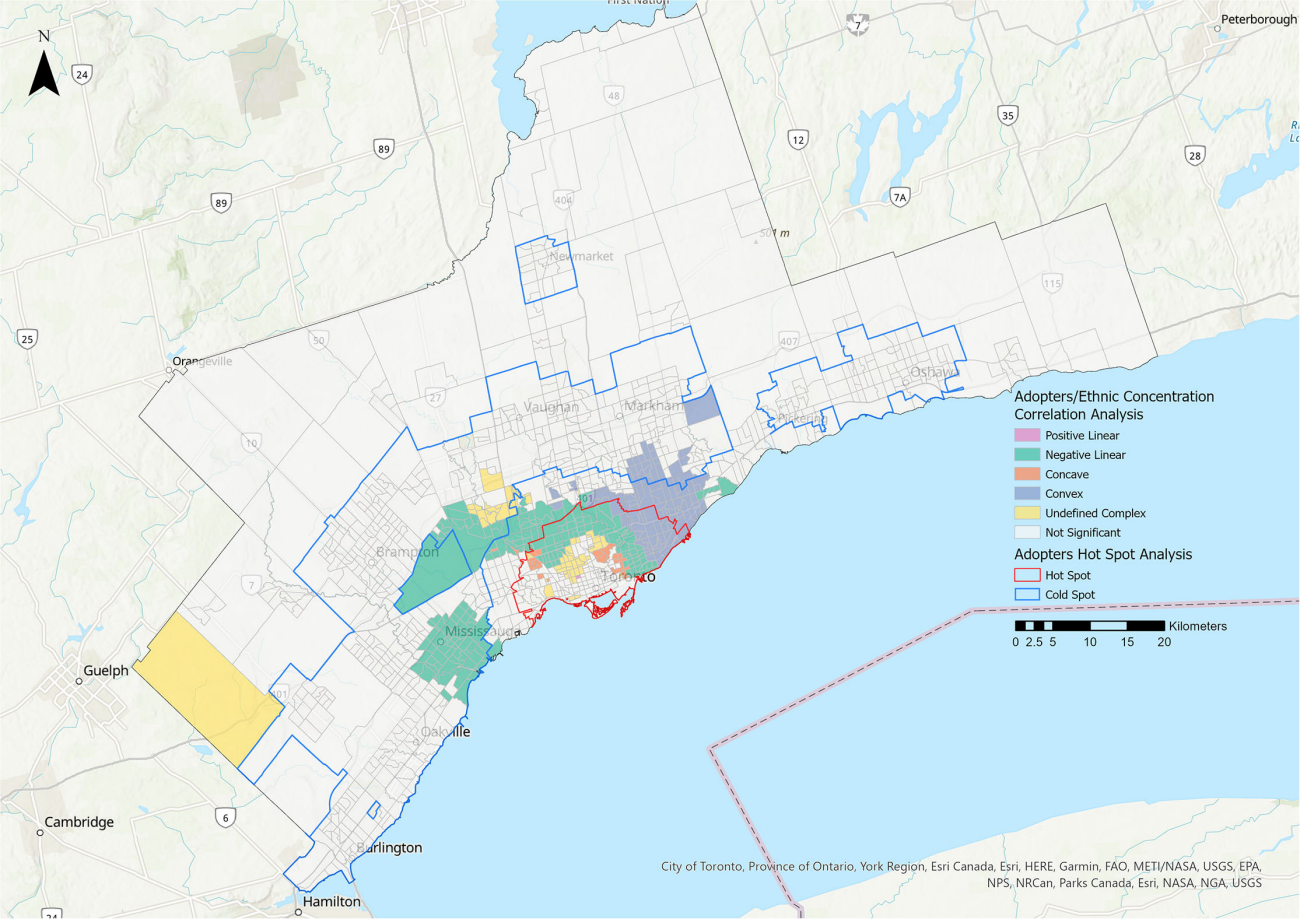
Correlation analysis for the adopters stakeholder group and the ethnic concentration metric of the 2016 Ontario Marginalization Index. Pink represents positive linear relationships, green represents negative linear relationships, orange represents concave relationships, blue represents convex relationships, and yellow represents undefined complex relationships.

Statistically significant relationships were identified between the adopters group and all five metrics (Table 3, Figures 6–8). More CTs had a positive linear relationship for the residential instability and the Indigenous metrics (15.38 and 16.37%, respectively) compared with the other relationship types (Figure 6, Table 3). The most prominent relationship for ethnic concentration was negative linear, representing 14.99% of CTs (Figure 8).

#### Surrenders

**Figure 9:**
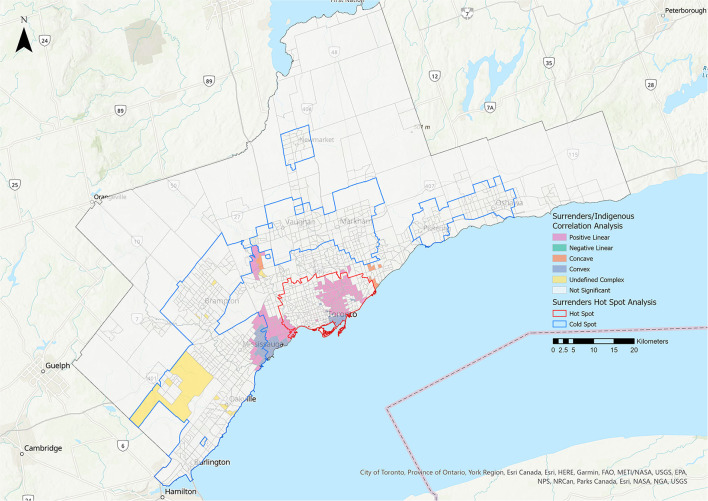
Correlation analysis for the surrenders stakeholder group and the rate of Indigenous residents metric. Pink represents positive linear relationships, green represents negative linear relationships, orange represents concave relationships, blue represents convex relationships, and yellow represents undefined complex relationships.

Statistically significant correlations were identified for surrenders and the Indigenous metric. These relationships were primarily positive linear (10.18%) (Figure 9, Table 3). No statistically significant relationships were identified between the surrenders group and the four ON-Marg metrics.

#### Public Veterinary Service Clients

**Figure 10:**
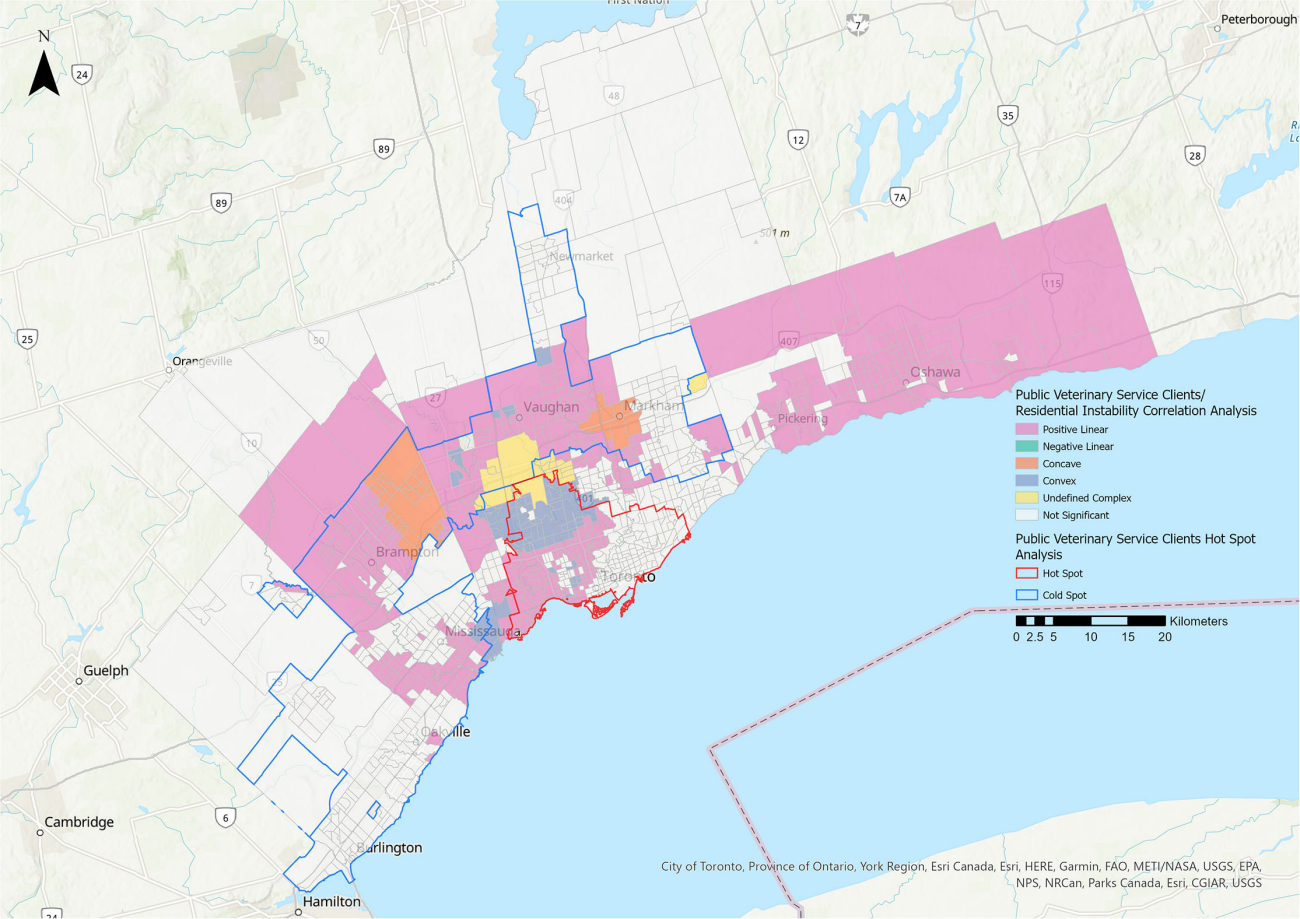
Correlation analysis for the public veterinary service clients stakeholder group and the residential instability metric of the 2016 Ontario Marginalization Index. Pink represents positive linear relationships, green represents negative linear relationships, orange represents concave relationships, blue represents convex relationships, and yellow represents undefined complex relationships.

**Figure 11:**
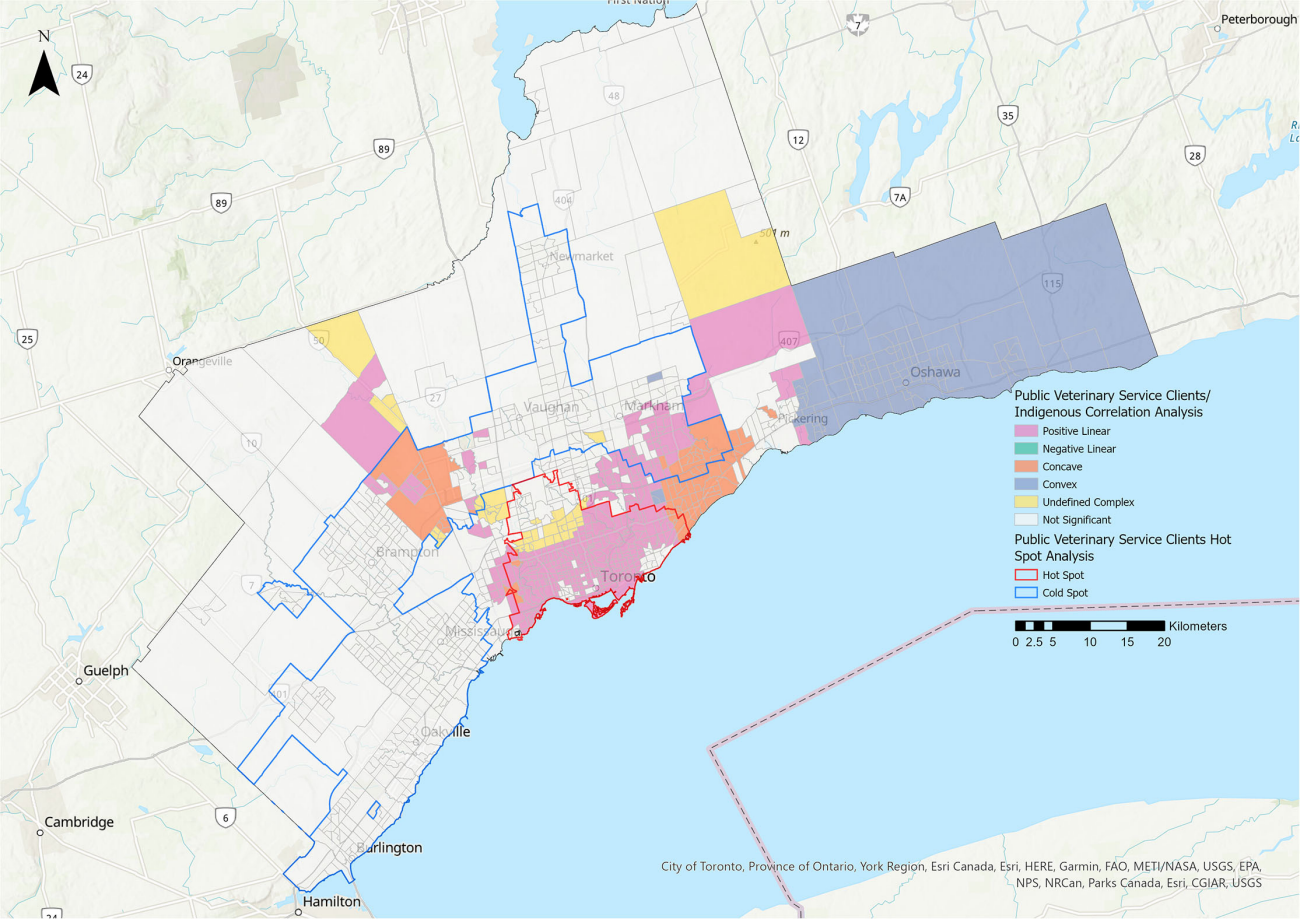
Correlation analysis for the public veterinary service clients stakeholder group and the Indigenous residents metric. Pink represents positive linear relationships, green represents negative linear relationships, orange represents concave relationships, blue represents convex relationships, and yellow represents undefined complex relationships.

Statistically significant relationships were identified between the PVS client group and the residential instability, material deprivation, and Indigenous metrics (Figures 10, 11, Table 3). The majority of the relationships identified with the residential instability metric were positive linear (39.95% of CTs). For the material deprivation metric, 11.15% of CTs were classified as positive linear relationships and for the Indigenous metric, 28.35% of CTs were positive linear. There were no statistically significant relationships for PVS clients and the dependency or ethnic concentration metrics.

#### Volunteers

The ethnic concentration metric identified a small number of negative linear relationships (0.55% of CTs) within the hot spot in Toronto, and convex relationships (1.33% of CTs) in the cold spots west of Toronto near Mississauga (Table 3). No other significant relationships were found.

#### Foster Parents

No statistically significant relationships were identified between foster parents and the five tested metrics.

## Discussion

This analysis was able to identify important relationships between stakeholder groups and socioeconomic metrics. The study fills an important gap in the literature pertaining to stakeholder use of AWO services and contributes to an understanding of Canadian animal welfare equity issues. Some of the relationships identified could be used to inform future welfare efforts by both THS and other local AWOs. Our data suggest that future initiatives could include development of satellite locations or mobile clinics, both to better serve families already using these services at some distance from the facility, and to reach less well-served populations. Education, particularly in schools, could be targeted to communities with lower adoption numbers. Additionally, a full assessment of marketing activities could be completed to ensure stakeholders who need the services most are aware they exist and know how to access them. Marketing strategies and methods may need to change in order to reach those who are not currently being reached.

One of the primary limitations of this study was the confounding factor of distance from THS. This effect can be summarized by Tobler's first law of geography, that “everything is related to everything else, but near things are more related than distant things” (24). This is commonly conceptualized in relation to the friction of distance theory (the idea that moving across space requires the expenditure of energy) (25) and distance decay theory (the idea that interactions between two positions decrease as distance increases) (18). Individuals living further from THS are less likely to utilize its services, particularly if there is another accessible AWO nearby, and therefore may appear to reflect a population under-served by THS that may not truly be under-served. This is supported by the fact that that many of the cold spots identified in this analysis overlap with the location of other AWOs that are geographically closer (Figure 1). However, the extent of the hot spots, particularly for the PVS client stakeholder group, suggests that THS is successfully serving a substantial geographic area, with no under-served areas identified within a 10 km radius of the facility.

Another inherent limitation of this type of spatial analysis is the influence of the modifiable areal unit problem, which is the effect that the boundaries (census tracts) used to aggregate the stakeholder data points can have on the results of the research (26). The results of the analysis may have differed if the data had been aggregated to different spatial boundaries. However, testing was also performed with smaller boundary sizes (dissemination areas) and little variation was noted between the two sets of results.

For certain stakeholder groups, removal of duplicates resulted in a substantial decrease in sample size. This was largely due the removal of instances of a single stakeholder appearing multiple times within the same year - for example, an individual surrendering multiple animals during the same year. Had these duplicates not been removed, stakeholder counts in certain CTs would have been artificially inflated. Further data cleaning, to remove stakeholders outside the GTA and those with incomplete addresses, had a much smaller effect on sample sizes, with 0.1% of the volunteer stakeholder group, 4.3% each of the adopter and foster groups, 7.9% of the PVS group and 13.6% of the surrender group being unavailable for geocoding. This was well within the recommendation of ≥80% “clean” data for GIS datasets in the animal welfare context^6^.

This study found that increasing rates of residential instability were associated with higher rates of adopters (Figure 6). One possible reason for this is that many THS adopters may be families living in rental homes (a factor included in the metric). While existing U.S. research suggests that certain housing factors such as renting correlate with lower pet ownership rates, this is most frequently due to landlord refusal to allow pets (27). Laws prohibiting Ontario landlords from banning pets (28) could contribute to the relationship identified in our study. In addition, the facility's shift to an Adopters Welcome framework^7^, some years ago, appears to have successfully decreased barriers to adoption. Further, the residential instability metric considers a greater number of children and total number of individuals living in the home to increase instability. While this may increase the residential instability metric, a greater number of children and total number of individuals living in the home generally correlates with an increase in pet ownership (29).

There was a predominantly negative linear relationship between ethnic concentration and adoption rates in central Toronto (Table 3, Figure 8). A convex relationship was also quite prominent, suggesting that while adopter rates largely decreased as ethnic concentration increased, in certain areas adopter rates then began to increase as ethnic concentration reached its highest levels. The ethnic concentration metric reflects both the proportion of residents who are recent immigrants and those who identify as part of a racialized community. A high percentage of GTA residents identify as racialized people or immigrants (51.4 and 46.1%, respectively) (15). One possible explanation for the relationship identified in the study may be cultural differences in pet ownership. Among 60 global societies, dogs were recognized as non-working companions or pets in only 22 and cats in 11 (30). Instead, animals have primarily working tasks such as hunting or vermin control (30). Alternative or parallel explanations might be lack of information, implicit bias during the adoption process, language barriers and financial considerations. A clearer understanding of the relevant factors would inform future efforts to address this service gap. Programs aimed at immigrant families could stress the benefits of pet ownership for reduction of stress for children (31), reduced feelings of loneliness and social isolation (32), increased socialization through community engagement (33), and as mental health supports (34).

In 4.08% of CTs, a higher material deprivation score was associated with a decrease in adopter rates (Figure 7). This was consistent with previous findings that higher income levels are correlated with higher pet ownership rates (35, 36). Adoption from AWOs may also be affected by factors such as physical and financial access, and perception by stakeholders. In one study, residents earning less than $20,000 per year were significantly more likely than higher income level groups to have acquired their pet from a family member or someone they knew, rather than from an AWO (37).

A legitimate concern for AWOs is the inadvertent transfer of animals from families facing material deprivation, through surrender, to higher income families, through adoption. Our analysis did not find any evidence of this. However, the largely positive linear relationship between surrenders and the proportion of Indigenous residents was noteworthy (Figure 9). This may be due to the fact that Indigenous people living in urban areas experience a higher rate of poverty (24%) than non-indigenous residents (13%) (15). Research has shown that, among low-income residents surrendering their pets in the U.S., costs (specifically those associated with veterinary care and food) were the most common reasons for surrendering an animal (38). In a 2017 Statistics Canada survey, 39% of Indigenous residents living in urban areas stated that they could not afford to pay an unexpected cost of $500 or more (39).

There were positive linear relationships between PVS clients and the residential instability, material deprivation and Indigenous metrics, with the largest effect for residential instability (39.95% of CTs) (Figure 10). The hot spot for PVS clients also extended further than other hot spots. These findings suggest that THS' public veterinary care programs are successfully reaching many families in need. Census tracts with a higher proportion of Indigenous residents were also associated with an increase in PVS use (Figure 11). This relationship may be explained by subsidized preventative wellness and spay/neuter services that are offered to residents with a Certificate of Indigenous Status and suggests that this approach is successful in making veterinary care more accessible to Indigenous individuals living within the GTA.

Notably, there were no correlations between PVS clients and ethnic concentration. This could in part be due to the lower rate of adopters associated with the ethnic concentration metric. Our findings were also in agreement with U.S. findings that race and ethnicity were not the primary determinants of veterinary care use in under-served communities (40). The authors hypothesized that structural barriers such as accessibility, transportation, and cost, rather than cultural barriers, could be more important drivers of lower access to veterinary care. However, U.S. surveys have also shown that race and ethnicity do have an effect on national pet ownership levels (41). A greater focus on culturally competent practices, targeted messaging and an understanding of accessibility barriers could allow AWOs to reach a greater proportion of families in need.

Analysis of the volunteers stakeholder group yielded very small negative linear and convex relationships with the ethnic concentration metric (see Table 3). The foster parents stakeholder group did not produce any significant relationships with any of the tested metrics. Little data is available regarding the characteristics of animal shelter volunteers or foster parents. A recent study of animal shelter volunteers in Michigan, US, found that most were white (68%), female (83%), had at least some post-secondary education (90%) and were employed full-time or retired (58%) (42). In contrast to the relatively homogenous pattern in that study, our data suggest that THS volunteers and foster parent groups were more representative of the broader community. The volunteers and foster parents stakeholder groups were substantially smaller than the other three groups, and this may also account for the lack of identified relationships. Volunteering for AWOs provides rewarding and meaningful engagement opportunities (42), and recruitment efforts should not be limited by assumptions about which segments of the community might be most interested or available. As most volunteers are recruited directly by the organization or through personal contacts (43), marginalized communities or groups should be actively approached.

This research into the spatial distribution of THS stakeholder groups identified many areas that are well served, as well as areas that are currently under-served. Correlation analysis identified many statistically significant relationships between the spatial distribution of stakeholder groups and the On-Marg Index and Indigenous metrics, such as a decrease in adopters as the ethnic concentration metric increased, and an increase in surrenders as the Indigenous metric increased. It should be noted that the relationships identified between the stakeholder groups and the five tested socioeconomic metrics do not necessarily indicate a causal relationship. Other confounding factors, such as variations in pet ownership with population density, may also influence stakeholder distributions.

Studies of this nature can allow AWOs to make informed decisions regarding their stakeholders, that take into account factors such as race, ethnicity, and socioeconomic status. Ultimately, this will promote a more equitable and inclusive environment for AWOs to better serve their communities and actively address systemic barriers to access.

Future research could analyze the geographic area closest to THS in more detail, as well as GTA neighborhoods designated as high priority due to socioeconomic factors. Analysis of stakeholder data from multiple AWOs within the GTA would also help gain a better understanding of the spatial distribution of stakeholders in the GTA as a whole. This could identify areas not being adequately served by any AWO.

Given its relative simplicity, the spatial analysis performed in this study could be replicated by geospatial data analysts from other AWOs hoping to evaluate the reach and inclusiveness of their services. Organizations increasingly collect large amounts of electronic data, which lends itself to novel forms of analysis. Animal welfare organizations should consider adding data analytics skills to their staff or volunteer bases. This would allow organizations to better understand metrics that currently are not commonly utilized in this sector.

## Data Availability Statement

The datasets presented in this article are not readily available because the raw dataset contains identifying information for animals and stakeholders. Requests to access the datasets should be directed to tbuckingham@torontohumanesociety.com.

## Author Contributions

All authors have made a substantial, direct, and intellectual contribution to the work and approved the final version.

## Funding

This study was generously supported by the Animal Welfare Foundation of Canada.

## Conflict of Interest

All authors were employed or contracted by THS during the study. The authors declare that the research was conducted in the absence of any other commercial or financial relationships that could be construed as a potential conflict of interest.

## Publisher's Note

All claims expressed in this article are solely those of the authors and do not necessarily represent those of their affiliated organizations, or those of the publisher, the editors and the reviewers. Any product that may be evaluated in this article, or claim that may be made by its manufacturer, is not guaranteed or endorsed by the publisher.

## Footnotes

^1^Examples of human-animal bond support statements: Toronto Humane Society (https://www.torontohumanesociety.com/purpose/human-animal-bond/), American Pets Alive! Human Animal Support Services (https://www.humananimalsupportservices.org/).

^2^Companions and Animals for Reform and Equity (CARE) (https://careawo.org/).

^3^Finch L. Best Friends Staff Open Up About Their Experiences with Racism in Animal Welfare. Best Friends blog. Available online at: https://network.bestfriends.org/tools-and-information/programs-spotlight/staff-experiences-racism-animal-welfare

^4^Black Lives Matter: racism in animal rescue—The Sniff. Available online at: http://www.thesniff.com/blm/

^5^ https://desktop.arcgis.com/en/arcmap/10.3/tools/spatial-statistics-toolbox/what-is-a-z-score-what-is-a-p-value.htm#ESRI_SECTION1_2C5DFC8106F84F988982CABAEDBF1440

^6^ASPCA. Targeting Risk: Preparing to Use GIS to Save More Lives. Available online at: https://www.aspcapro.org/sites/default/files/gis_targeting_risk_webinar_slides.pdf

^7^Humane Society of the United States. Adopters Welcome: Finding, Engaging and Supporting More Adopters. Available online at: https://humanepro.org/page/adopters-welcome-manual

## Supplementary Material

Supplementary Figure 1 Map showing the number of stakeholders per census tract for the adopters stakeholder group.

Supplementary Figure 2 Map showing the number of stakeholders per census tract for the surrenders stakeholder group.

Supplementary Figure 3 Map showing the number of stakeholders per census tract for the public veterinary service clients stakeholder group.

Supplementary Figure 4 Map showing the number of stakeholders per census tract for the volunteers stakeholder group.

Supplementary Figure 5 Map showing the number of stakeholders per census tract for the foster parents stakeholder group.

Supplementary Figure 6 Map showing the normalized (number of stakeholders per 1,000 people residing in each census tract) data for the adopters stakeholder group.

Supplementary Figure 7 Map showing the normalized (number of stakeholders per 1,000 people residing in each census tract) data for the surrenders stakeholder group.

Supplementary Figure 8 Map showing the normalized (number of stakeholders per 1,000 people residing in each census tract) data for the public veterinary service clients stakeholder group.

Supplementary Figure 9 Map showing the normalized (number of stakeholders per 1,000 people residing in each census tract) data for the volunteers stakeholder group.

Supplementary Figure 10 Map showing the normalized (number of stakeholders per 1,000 people residing in each census tract) data for the foster parents stakeholder group.
