# Supplementary figures and images for: Where Are We on the Animal Welfare Map? Using GIS to Assess Stakeholder Diversity and Inclusion
Source: Front Vet Sci. 2021 Dec 13;8:785071. doi: 10.3389/fvets.2021.785071 (PMC8710581; doi:10.3389/fvets.2021.785071)

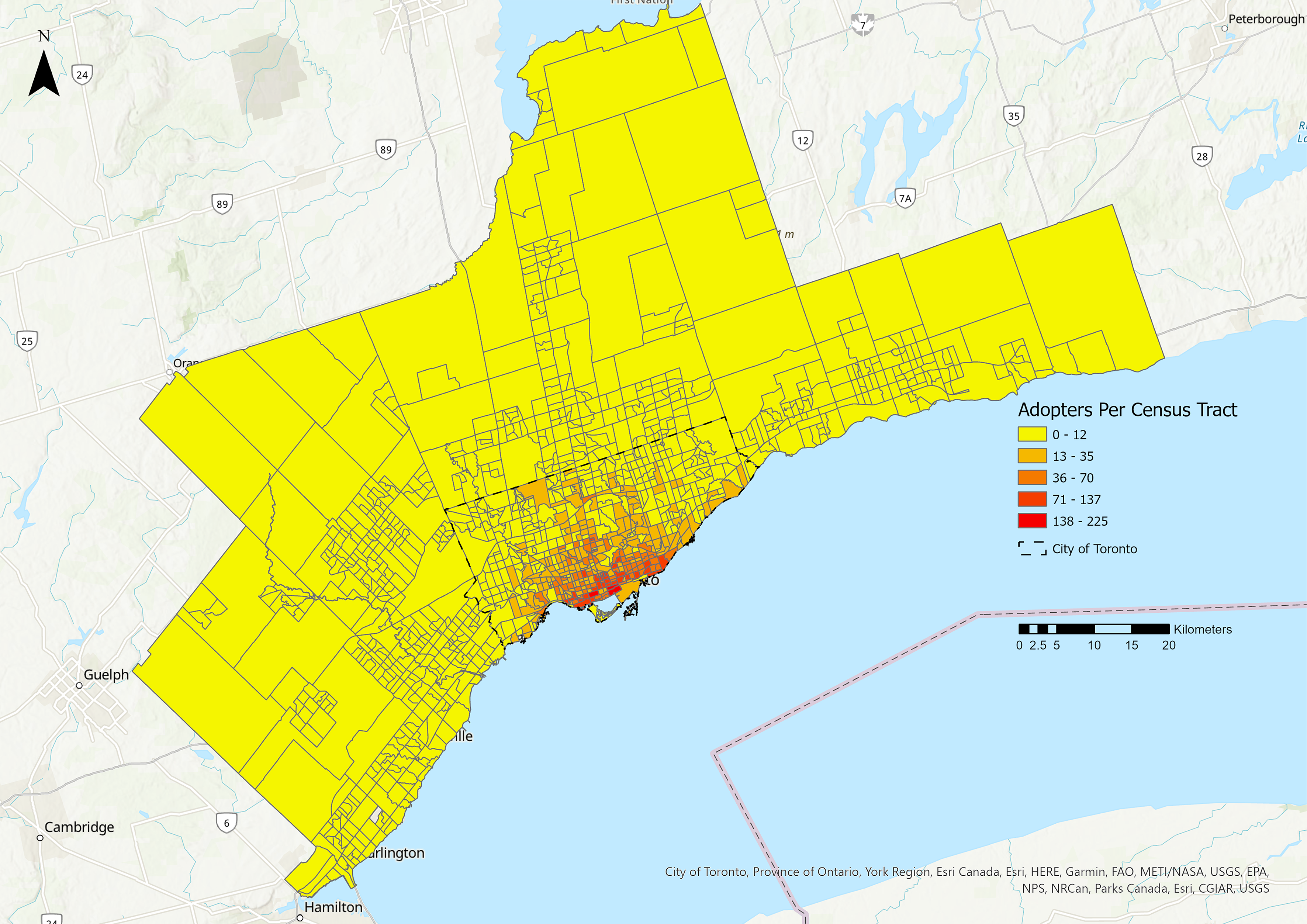

Supplement: Supplementary Figure 1 — Map showing the number of stakeholders per census tract for the adopters stakeholder group. [file Image_1.TIFF]

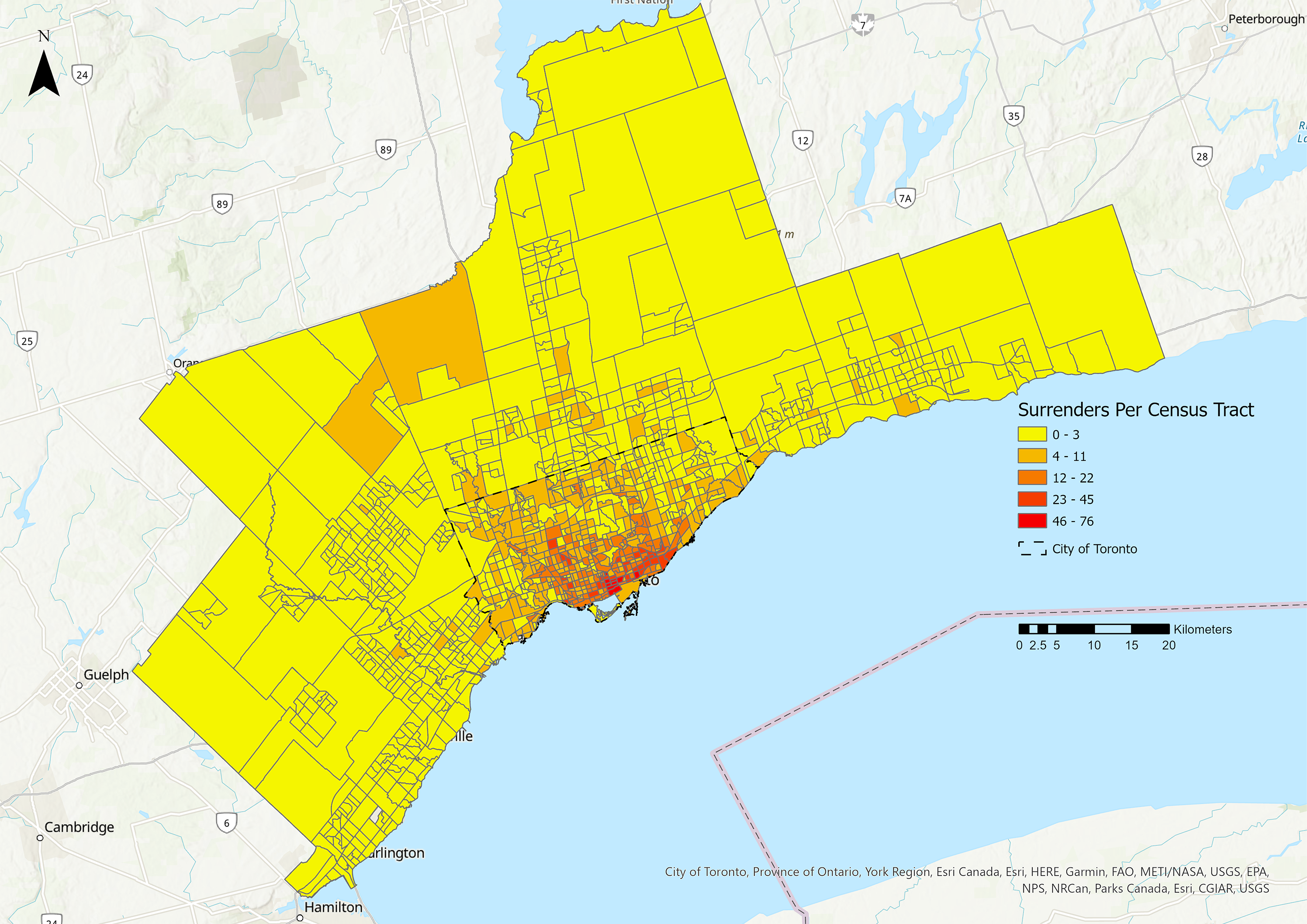

Supplement: Supplementary Figure 2 — Map showing the number of stakeholders per census tract for the surrenders stakeholder group. [file Image_2.TIFF]

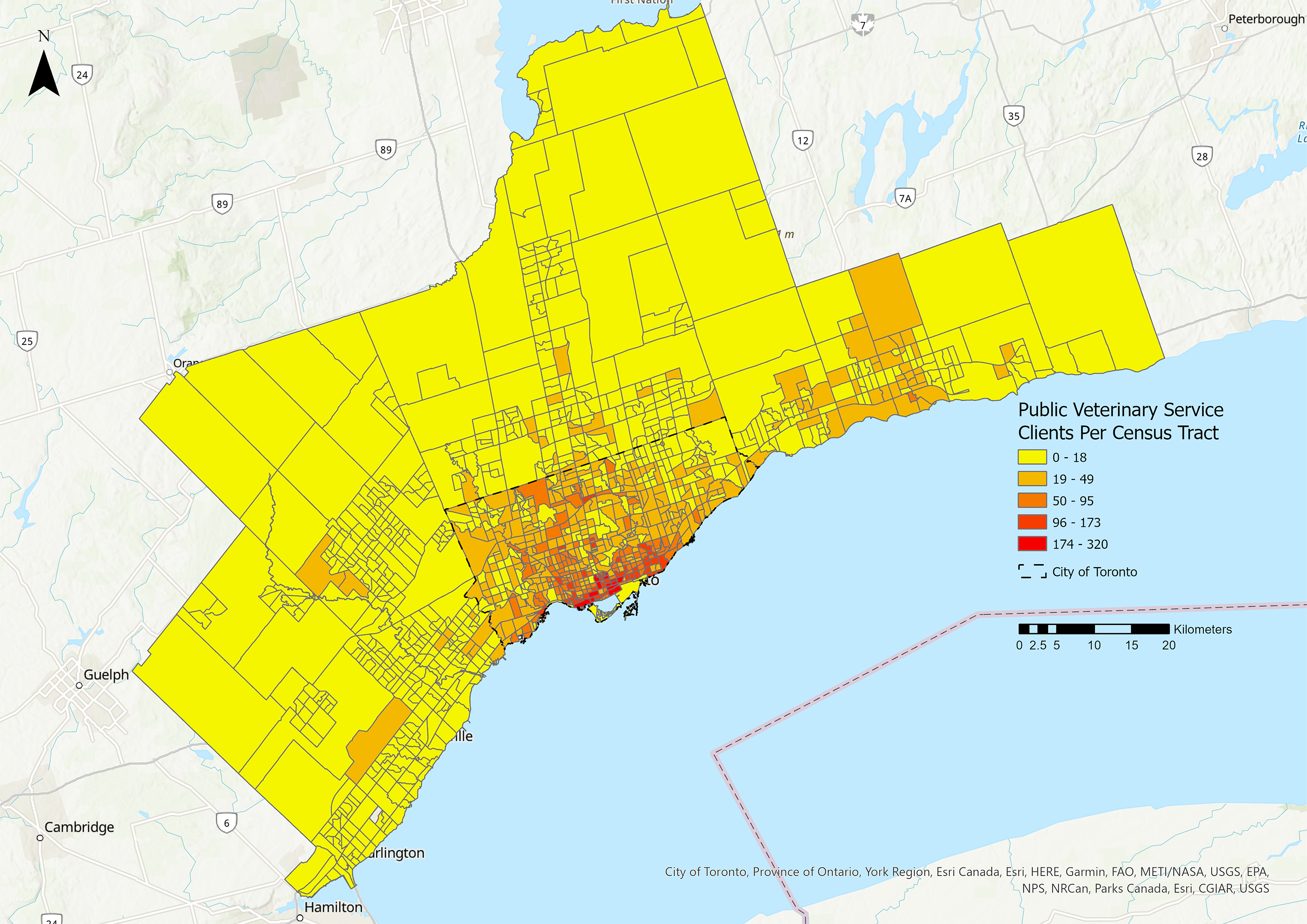

Supplement: Supplementary Figure 3 — Map showing the number of stakeholders per census tract for the public veterinary service clients stakeholder group. [file Image_3.TIFF]

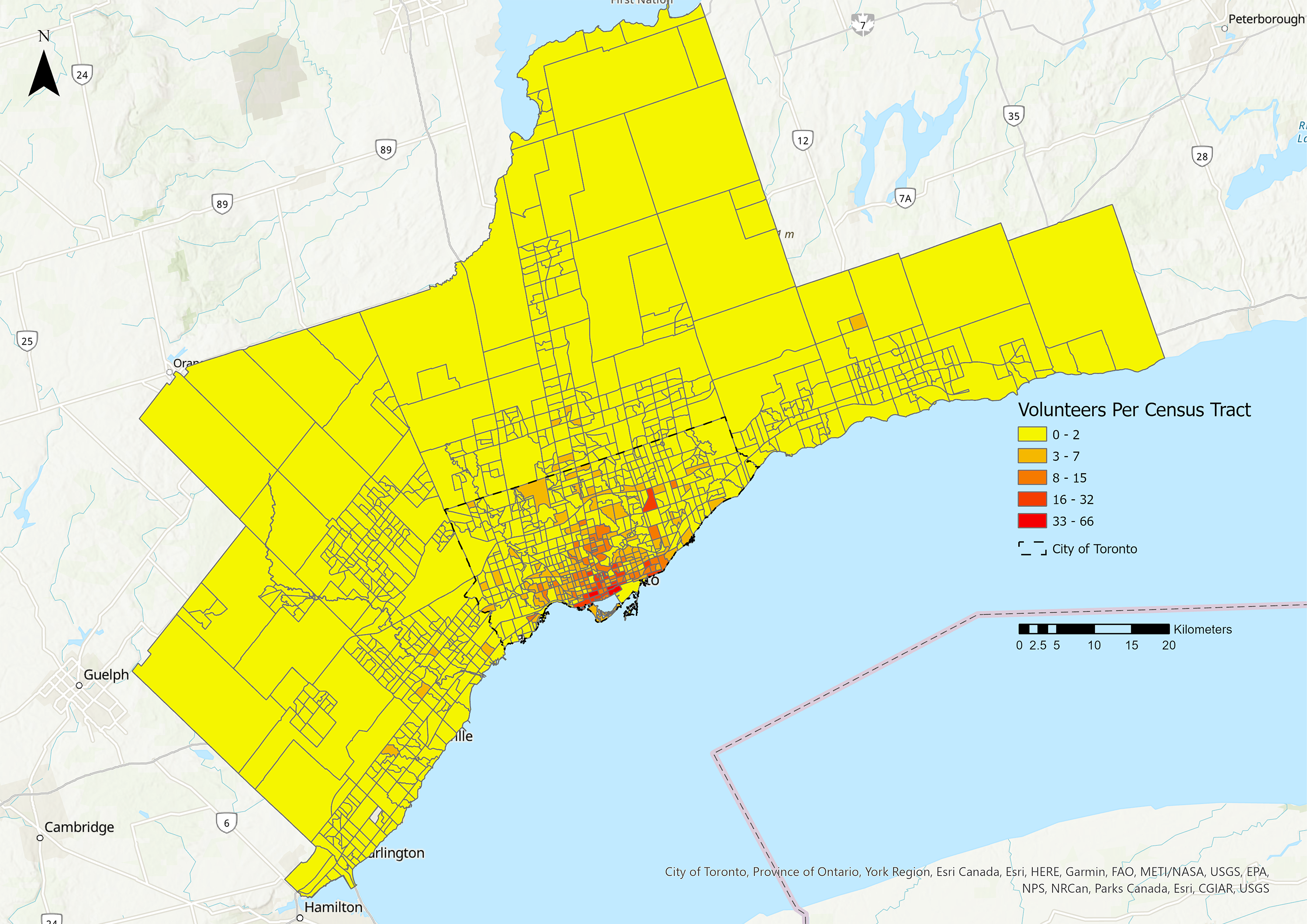

Supplement: Supplementary Figure 4 — Map showing the number of stakeholders per census tract for the volunteers stakeholder group. [file Image_4.TIFF]

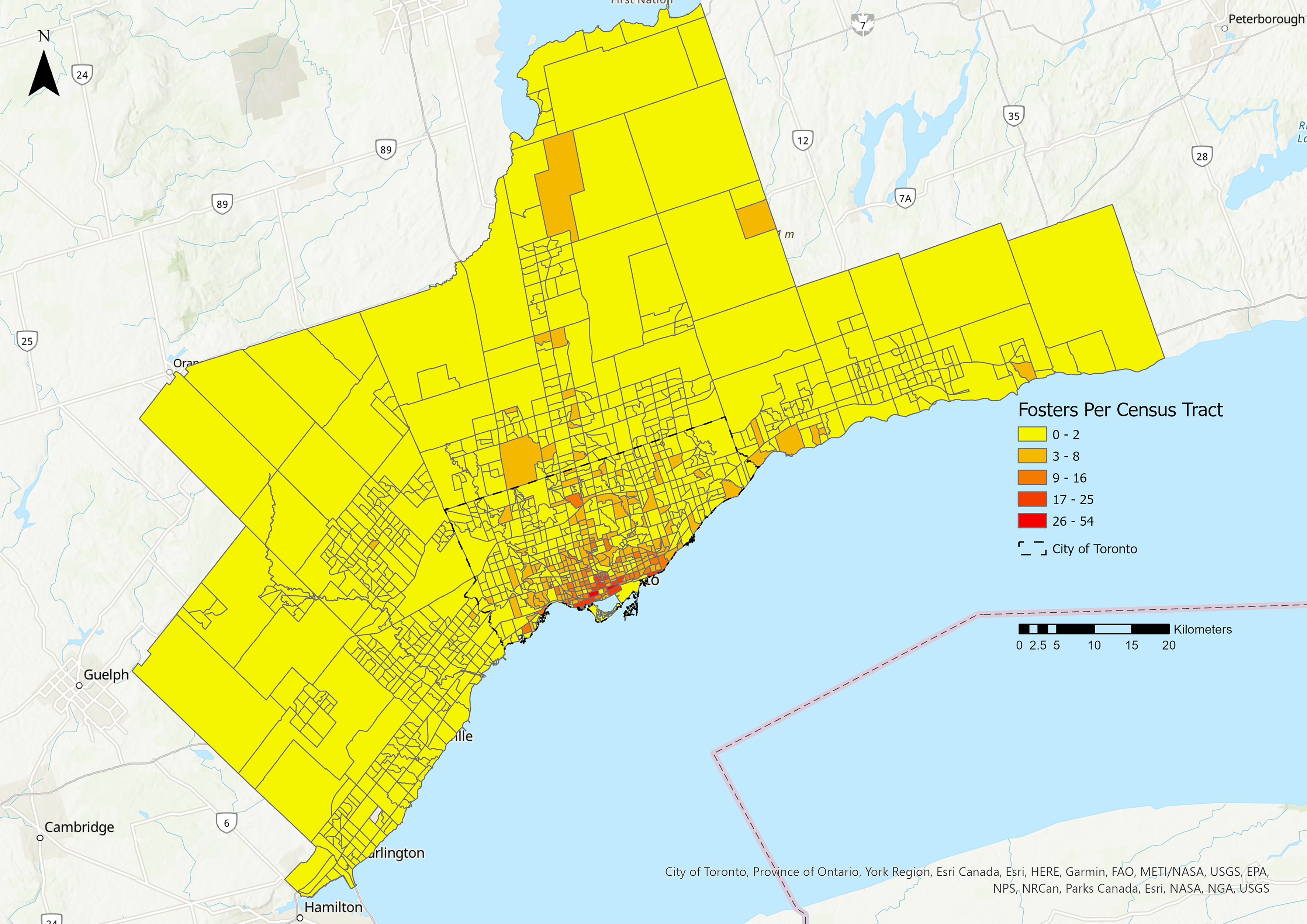

Supplement: Supplementary Figure 5 — Map showing the number of stakeholders per census tract for the foster parents stakeholder group. [file Image_5.TIFF]

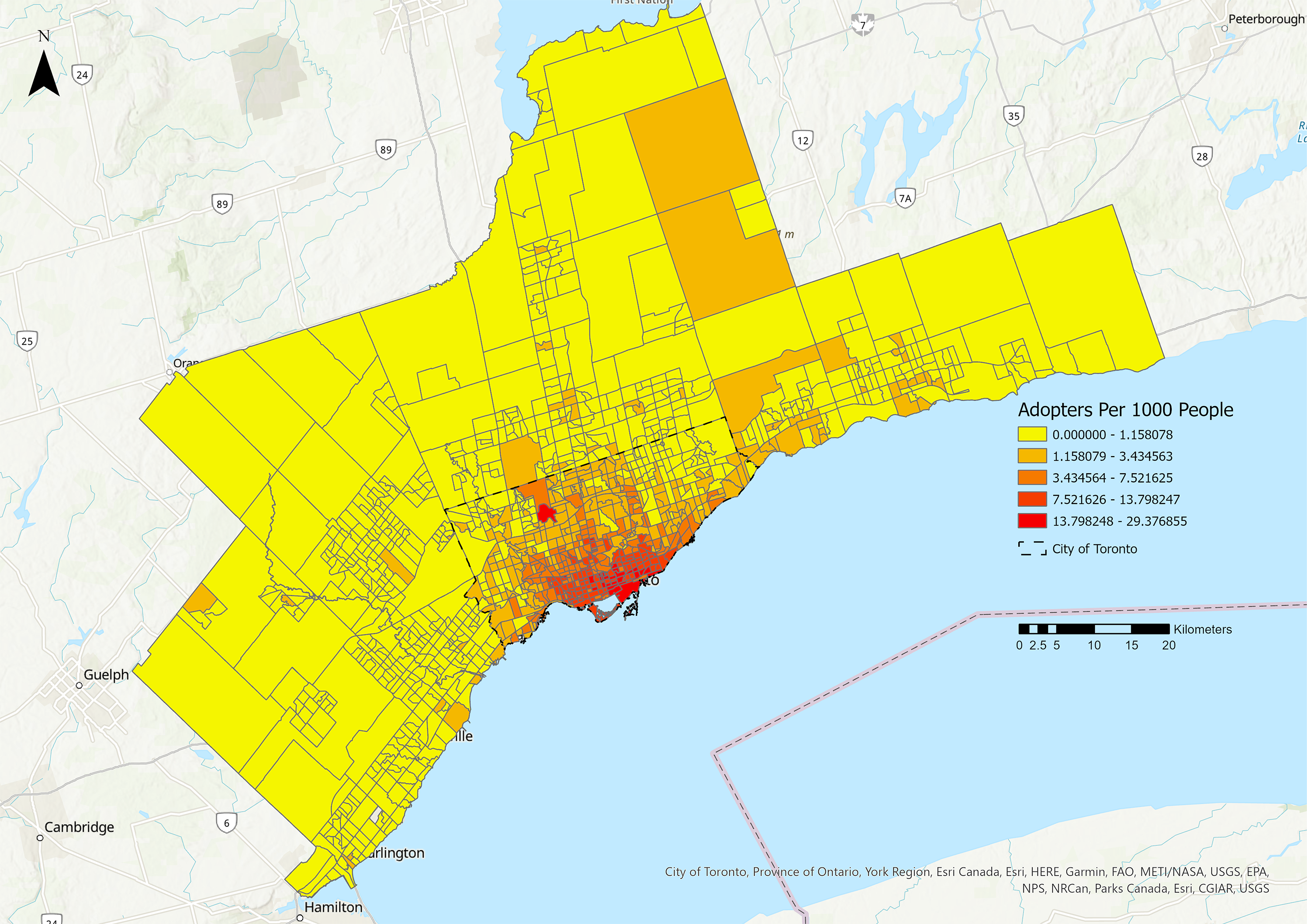

Supplement: Supplementary Figure 6 — Map showing the normalized (number of stakeholders per 1,000 people residing in each census tract) data for the adopters stakeholder group. [file Image_6.TIFF]

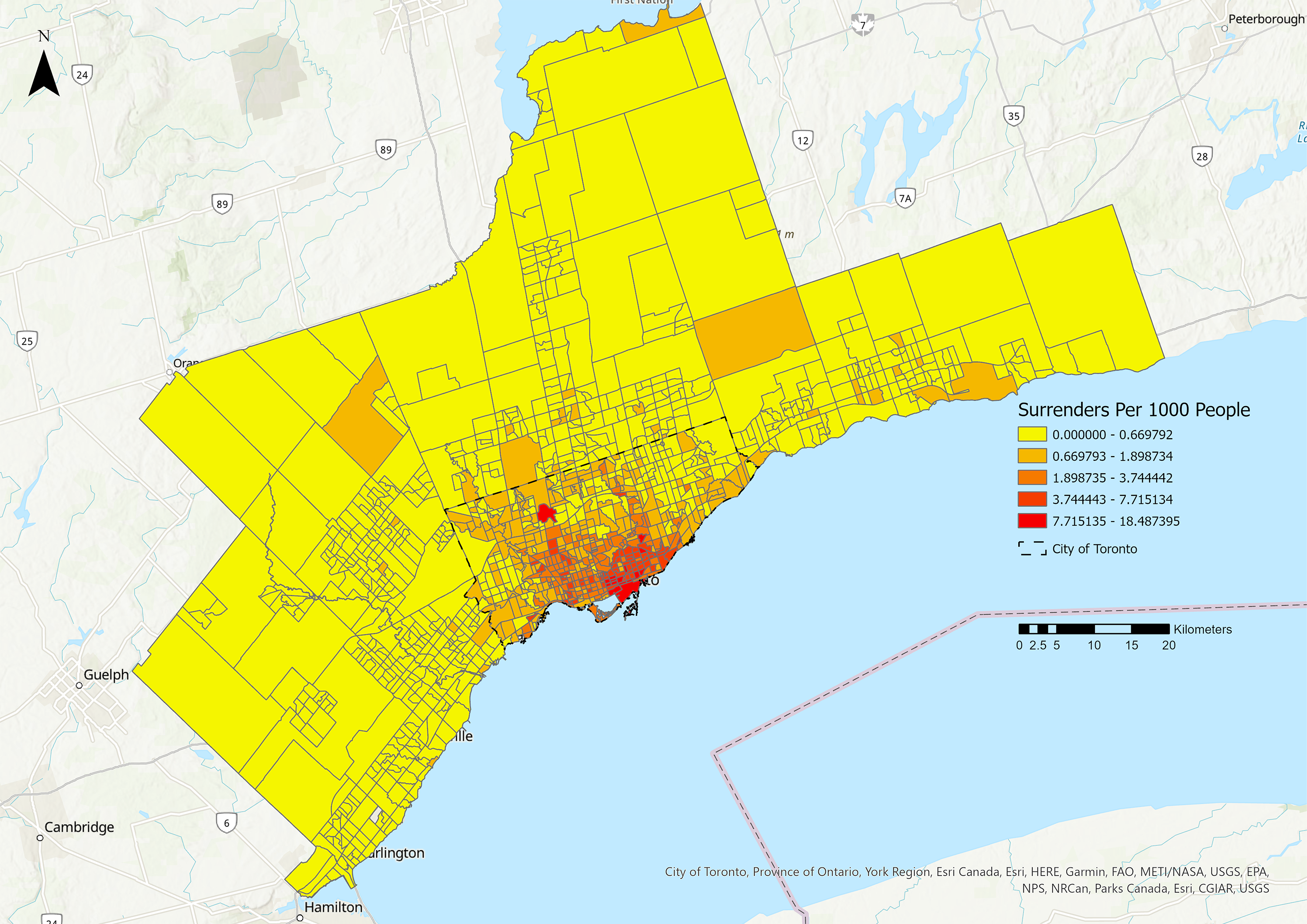

Supplement: Supplementary Figure 7 — Map showing the normalized (number of stakeholders per 1,000 people residing in each census tract) data for the surrenders stakeholder group. [file Image_7.TIFF]

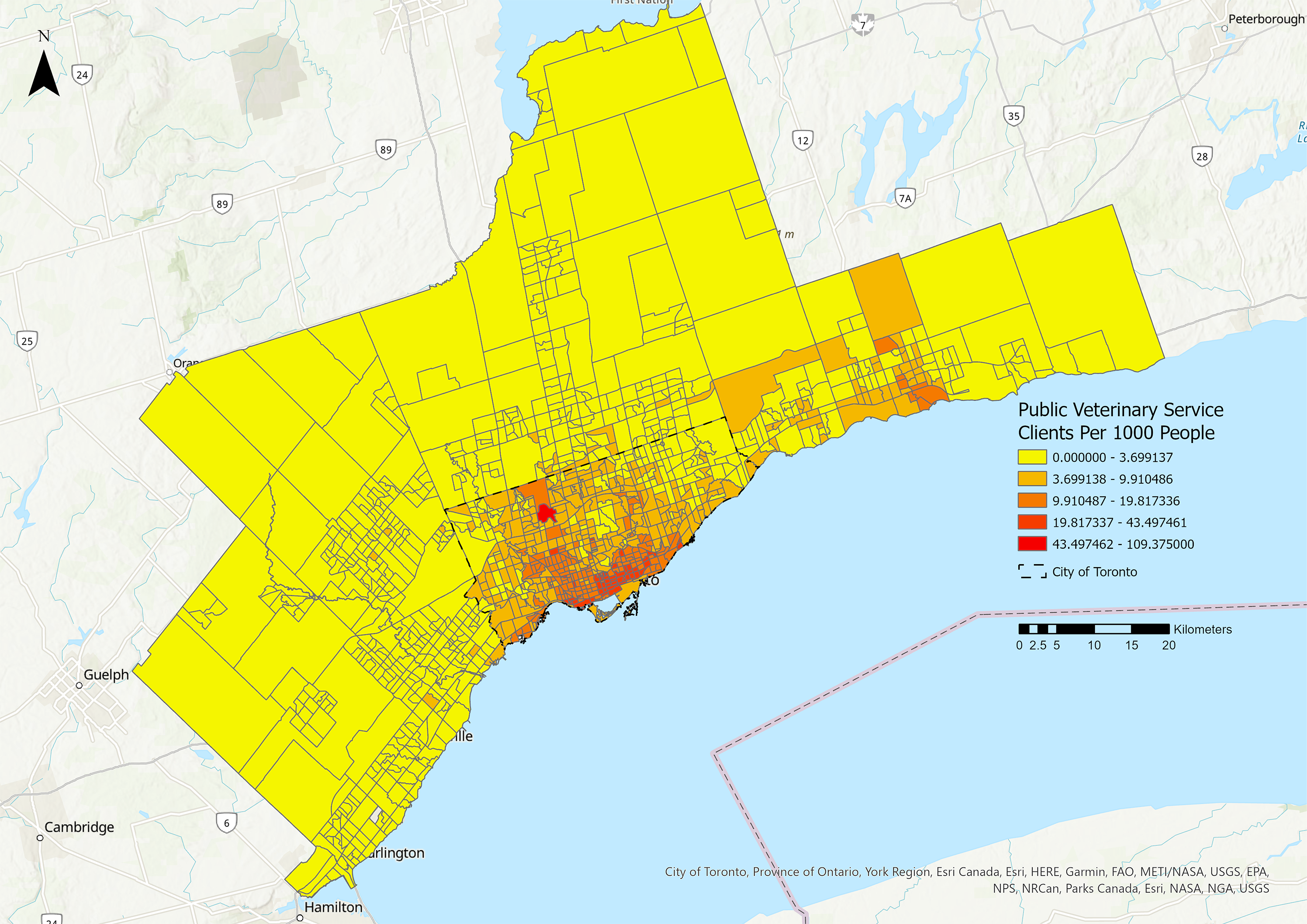

Supplement: Supplementary Figure 8 — Map showing the normalized (number of stakeholders per 1,000 people residing in each census tract) data for the public veterinary service clients stakeholder group. [file Image_8.TIFF]

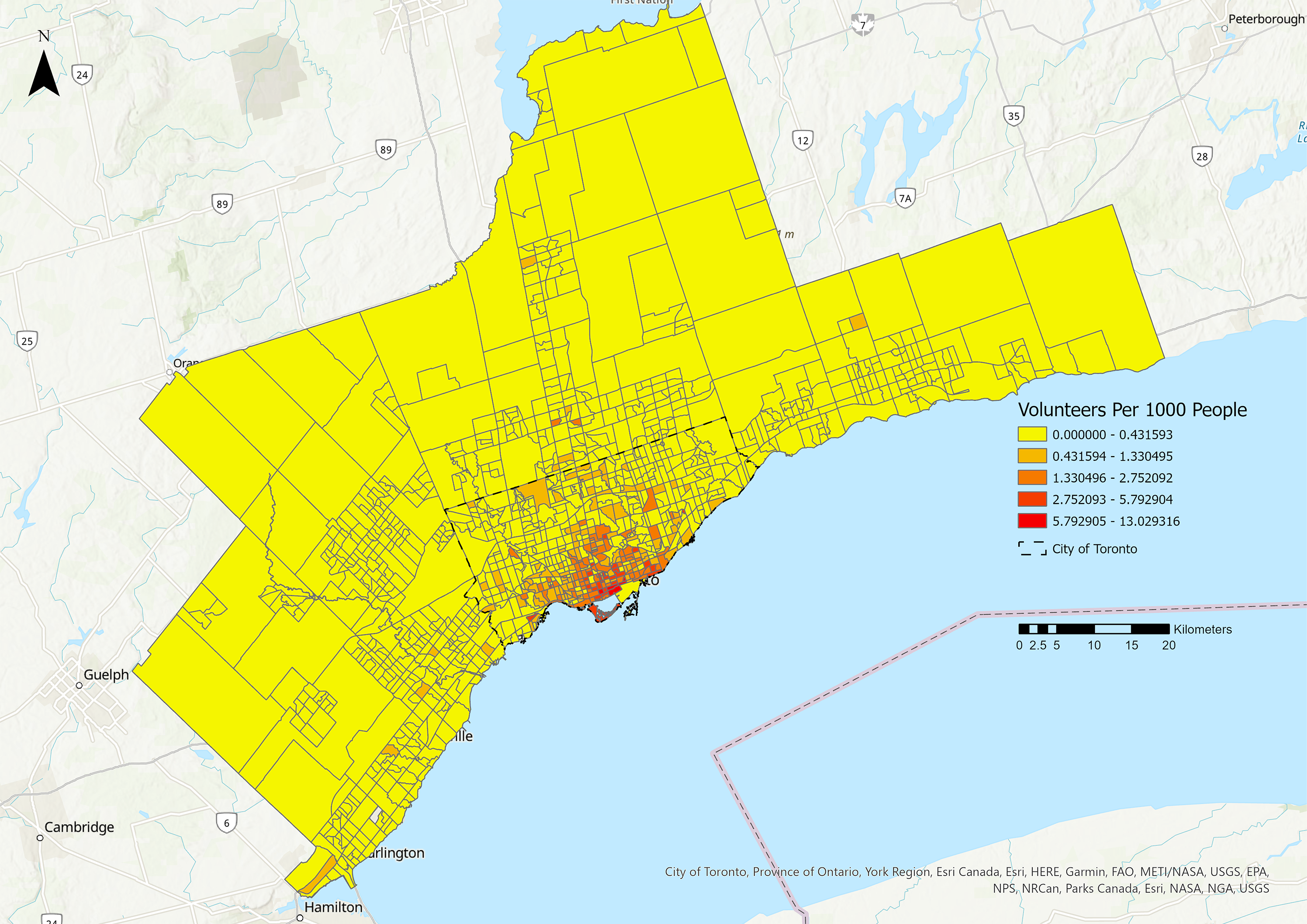

Supplement: Supplementary Figure 9 — Map showing the normalized (number of stakeholders per 1,000 people residing in each census tract) data for the volunteers stakeholder group. [file Image_9.TIFF]

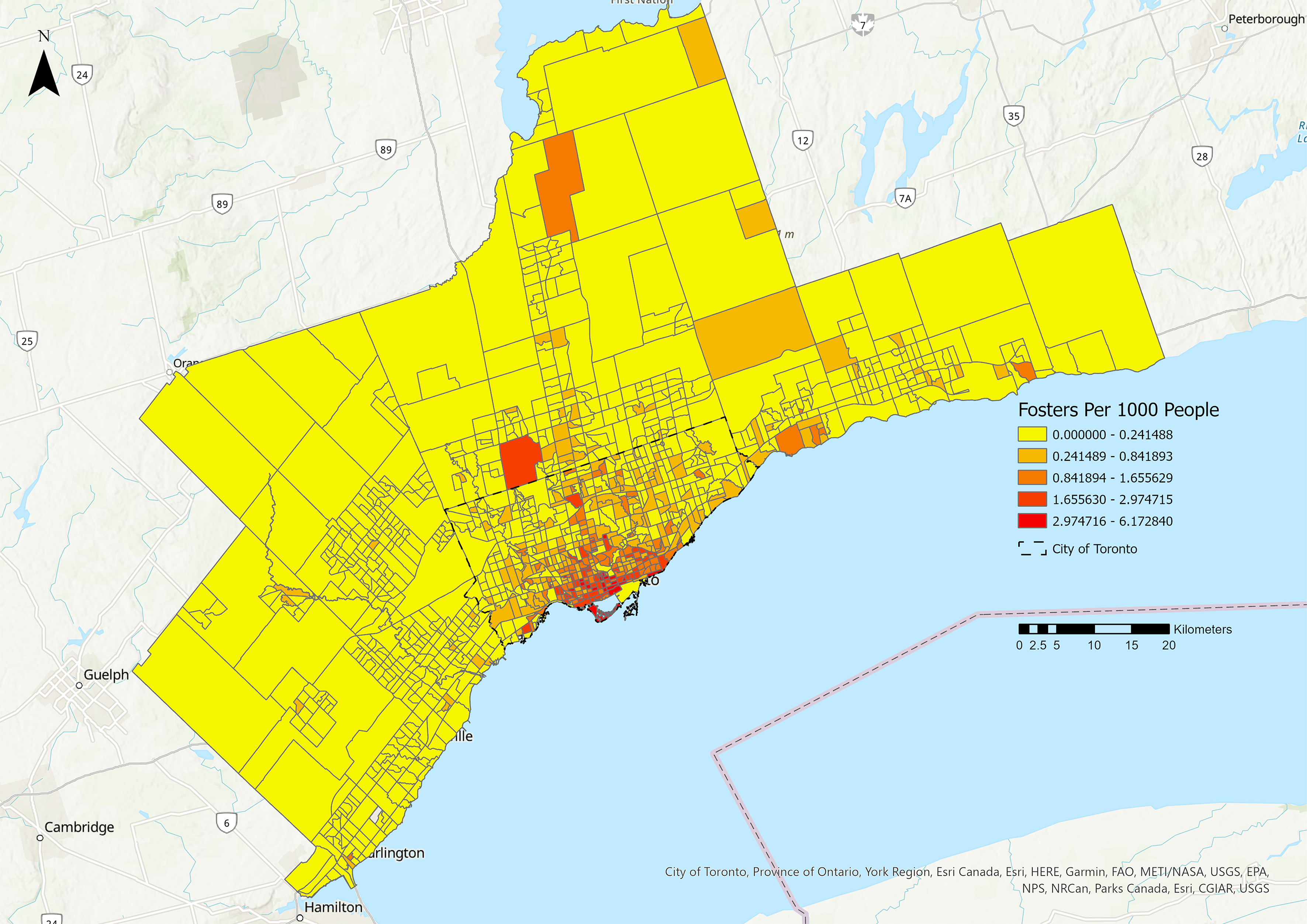

Supplement: Supplementary Figure 10 — Map showing the normalized (number of stakeholders per 1,000 people residing in each census tract) data for the foster parents stakeholder group. [file Image_10.TIFF]
